# The Co-Expression of Estrogen Receptors ERα, ERβ, and GPER in Endometrial Cancer

**DOI:** 10.3390/ijms24033009

**Published:** 2023-02-03

**Authors:** Marko Hojnik, Maša Sinreih, Maja Anko, Neli Hevir-Kene, Tamara Knific, Boštjan Pirš, Snježana Frković Grazio, Tea Lanišnik Rižner

**Affiliations:** 1 Institute of Biochemistry and Molecular Genetics, Faculty of Medicine, University of Ljubljana, 1000 Ljubljana, Slovenia; 2 Department of Pathology, University Medical Centre Maribor, 2000 Maribor, Slovenia; 3 Department of Gynecology, University Medical Centre, 1000 Ljubljana, Slovenia

**Keywords:** ERα, ERβ, GPER, immunohistochemistry, western blotting, qPCR, endometrial cancer

## Abstract

Estrogens have important roles in endometrial cancer (EC) and exert biological effects through the classical estrogen receptors (ERs) ERα and ERβ, and the G-protein–coupled ER, GPER. So far, the co-expression of these three types of ERs has not been studied in EC. We investigated ERα, ERβ, GPER mRNA and protein levels, and their intracellular protein distributions in EC tissue and in adjacent control endometrial tissue. Compared to control endometrial tissue, immunoreactivity for ERα in EC tissue was weaker for nuclei with minor, but unchanged, cytoplasmic staining; mRNA and protein levels showed decreased patterns for ERα in EC tissue. For ERβ, across both tissue types, the immunoreactivity was unchanged for nuclei and cytoplasm, although EC tissues again showed lower mRNA and protein levels compared to adjacent control endometrial tissue. The immunoreactivity of GPER as well as mRNA levels of GPER were unchanged across cancer and control endometrial tissues, while protein levels were lower in EC tissue. Statistically significant correlations of estrogen receptor α (*ESR1*) versus estrogen receptor β (*ESR2*) and *GPER* variant 3,4 versus *ESR1* and *ESR2* was seen at the mRNA level. At the protein level studied with Western blotting, there was significant correlation of ERα versus GPER, and ERβ versus GPER. While in clinical practice the expression of ERα is routinely tested in EC tissue, ERβ and GPER need to be further studied to examine their potential as prognostic markers, provided that specific and validated antibodies are available.

## 1. Introduction

Endometrial cancer (EC) is the fourth-most-common cancer in women in Western Europe and the USA, with the majority of cases arising in postmenopausal women [1,2]. EC can be classified into estrogen-dependent type 1 (80% of all cases) and the poorly differentiated, more aggressive type 2, which is traditionally considered as estrogen-independent [3,4]; however, several studies suggest that estrogens also have roles in EC type 2 [5,6,7]. This exposure to estrogens that is not opposed by progesterone or synthetic progestins increases the mitotic activity of endometrial cells, along with the number of DNA replication errors. This can lead to somatic mutations that result in a malignant phenotype [3,8].

More recently, the Cancer Genome Atlas (TCGA) project has discovered four molecular prognostic subtypes: ultramutated—defined by POLE mutations; microsatellite instable (MSI) hypermutated; copy-number-low/p53-wild-type (p53 wt); and copy-number-high/p53-mutated (p53mt). Tumors from each of the first three molecular subtypes have high expression levels of *ESR1* encoding ERα, while the copy-number-high/p53-mutated (p53mt) group shows no/low expression and is prognostically worse [9]. 

Estrogens exert their biological effects through the estrogen receptors (ERs) [10,11]. Both genomic and rapid (non-genomic) signaling events initiated by estrogens have traditionally been attributed solely to the classical ERs (i.e., ERα and ERβ) [12,13], as classical ERs can be postranslationally palmitoylated and anchored to the plasma membrane [14,15,16,17]. More recently, the G-protein–coupled ER, GPER (also known as GPR30), has been implicated in mediating the rapid responses of the estrogens [18,19,20,21].

In addition to the classical, slow genomic mechanisms of estrogen actions [12,22,23,24], estrogen receptor complexes also act through indirect genomic signaling by interacting with different proteins, other transcription factors, and response elements [12]. Due to the numerous possible combinations between ERs and co-activators and co-repressors, and the potential for these complexes to bind to different gene promoters, ERα and ERβ can have opposing actions. ERβ can also inhibit ERα activity by increasing ERα degradation [25]. ERα is considered to be the main receptor of E2, responsible for normal human development and reproduction [26,27], with important roles in development of different cancers such as breast, ovarian, colon, and endometrial cancer [28,29,30]. The less-well-described receptor ERβ has opposing actions on ERα function as studied in breast cancer, prostate cancer, gynecological cancers, and endocrine cancers; it is speculated to be a tumor suppressor [29,31,32,33]. However, several studies of ERβ in endometrial cancer showed contradictory results [27], and due to problems with the specificity of commercially available ERβ antibodies that scientific community have reported many times before [34,35,36], the results of these studies should be considered with caution. 

Membrane (m)ERs can have rapid non-genomic cellular responses that act via activation of protein kinase cascades and second-messenger production [22,37]. There is also growing evidence of genomic and non-genomic signaling crosstalk and ER ligand-independent signaling [12]. ERs have been detected in the plasma membrane of isolated endometrial cells [38] and in EC cell lines where estrogens act via the mitogen activated protein kinase (MAPK) signaling pathway and calcium influx [39,40,41] and via PKCα [38]. 

In EC in general, there are higher levels of ERα than ERβ [42,43,44,45,46,47,48]. In endometrial tissue, ERβ has important roles in normal homeostasis, cell turnover, and regeneration, furthermore it has a role in most benign and malignant endometrial diseases [27]. A shift in the ratio between ERα and ERβ has been suggested to be involved in endometrial carcinogenesis [42,43,44,45,46]. ERα expression is higher in the early stages of EC and becomes decreased in advanced EC [19,49,50,51]. The loss of expression of ERα in EC has been associated with stage, tumor grade, and lymph node involvement; however, only few studies have revealed an association with disease-free or overall survival [52,53,54,55,56]. High ERβ was associated with shorter disease-free survival in patients with EC and regional lymph node metastasis [57].

The activation of GPER leads to the activation of several signaling pathways involving epidermal growth factor receptor (EGFR), the MAPK/ extracellular regulated protein kinase (MAPK/ERK), the phosphatidylinositol 3-kinase/protein kinase B (PI3K/Akt), the protein kinase A (PKA), and the phospholipase C (PLC) pathways [18,20,58]. GPER also mediates an increase in the activity of endothelial nitric oxide (NO) synthase (eNOS), an increase in the activity of sphingosine kinase, and regulates calcium mobilization, potassium channels, and the gene expression of, e.g., c-fos and the cyclins A, D1, or E [59]. Rapid GPER-mediated responses often lead to tumor promotion [21]. Recently, the ability of GPER receptor to stimulate cancer progression by the regulation of miRNA expression has been discovered [60,61]. However, the localization of GPER in cells has not been determined unequivocally, with reports on GPER localization in the endoplasmic reticulum, Golgi apparatus, nucleus, and at the cell membrane [58,62,63]. 

In EC tissues, both elevated [59,64] and decreased [65] *GPER* expression have been reported, which have been correlated with disease progression [47,66,67,68], and in EC cell lines, activation of GPER has been shown to stimulate cell proliferation and invasion [64,69]. The loss of GPER expression predicts poor survival in patients with EC [66]. However, similarly to ERβ, many commercially available anti-GPER antibodies on the market are nonspecific; therefore, caution is warranted when interpreting results of these studies [34,35,36].

ERα status is an important prognostic marker in hormone-dependent cancers, and GPER has also been suggested to have the potential to predict disease progression in patients with EC. Despite numerous studies of ERs, their precise role and their interplay in EC is still not clear. The aim of the present study was thus to investigate the expression of ERα, ERβ, and GPER in EC and adjacent endometrial tissue and to analyze potential correlation between their expressions. We thus studied their tissue mRNA and protein levels, their intracellular protein distributions, and evaluated their co-expression.

## 2. Results

### 2.1. Lower mRNA and Protein Levels and IHC Scores of ERα in EC Tissues

**Figure 1:**
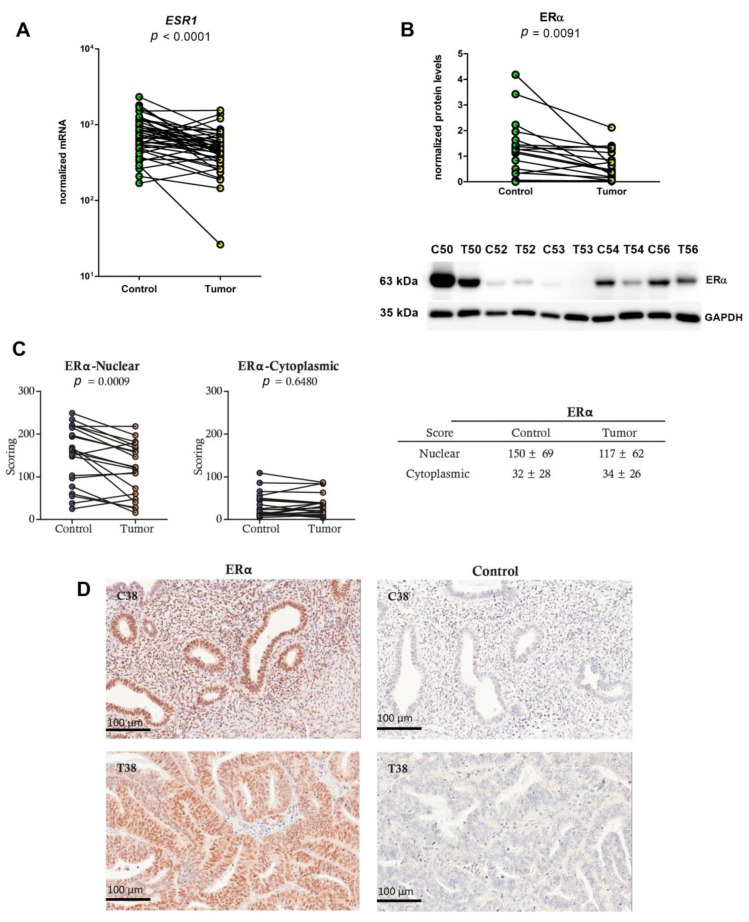
ERα mRNA and protein levels in endometrial cancer and adjacent control endometrium. (**A**) Before-and-after graph shows the normalized expression levels of the *ESR1* gene in adjacent endometrial tissue (Control) and the corresponding EC tissue (Tumor). The levels of gene expression are on a logarithmic scale. (**B**) ECL detection of ERα (63 kDa band). 18 paired samples were analyzed using anti-ERα antibodies (SP1, Thermo Fisher Scientific, Cat. #: RM-9101-15, Lot:9101513081A), GAPDH was used as a normalization control. Before-and-after plots show quantification of Western blotting data. Below, representative membrane with ERα and GAPDH staining is shown. EC tissue (T), adjacent control endometrial tissue (**C**). (**C**) IHC scores in 21 samples from adjacent control endometrial tissue (Control) and EC tissue (Tumor). Table shows mean scores ± standard deviations, while before-and-after graphs show nuclear and cytoplasmic ERα staining (anti-ERα antibodies, 1D5 Dako, Cat. #: M7047, lot 1: 00034057, lot 2: 20015818). (**D**) IHC staining in representative paired adjacent control endometrial tissue (C38) and EC tissue (T38) for ERα. In the negative controls (Control), the primary antibodies were replaced with serum of the same animal species (rabbit or mouse). Both anti-ERα antibodies were validated (Supplementary Figure S4 and Table S5).

ERα gene (*ESR1*) expression was evaluated at the mRNA and protein levels in our previous study [70], however, here we further investigated its expression on a larger group of samples. We confirmed the lower level of *ESR1* mRNA by qPCR in 44 paired samples of cancer as compared to control endometrial tissue and lower protein levels of ERα by Western blotting with antibodies SP1 in 18 paired samples. In both cases, the difference was statistically significant (Figure 1A, *p* < 0.0001, Figure 1B, *p* = 0.0091). Statistical analysis using two-way ANOVA showed that menopausal status and tumor grade do not affect *ESR1* expression at the mRNA or protein levels. 

We next performed IHC analysis with antibodies 1D5 in 21 specimens that included both EC tissue and the adjacent control endometrial tissue (Figure 1C,D). ERα was detected predominantly in epithelial cells, and to a lesser extent also in stromal cells, and the staining was stronger in the nuclei than in the cytoplasm. Lower IHC scores for nuclear staining of cancerous glands were observed in 15 of 21 specimens, and the differences between the EC tissue and the control adjacent endometrial tissue were statistically significant (*p* = 0.0009). On the other hand, no differences were seen in the cytoplasmic staining for ERα between the cancerous and the control endometrial glands. 

**Figure 2:**
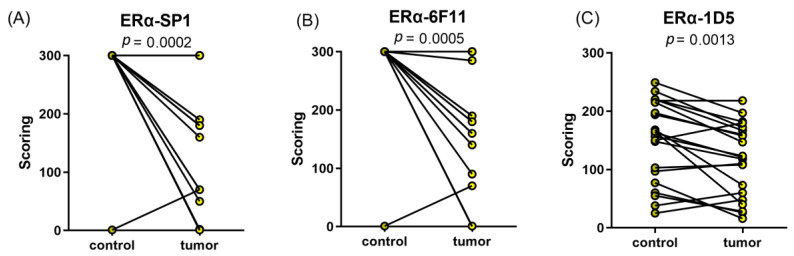
Comparison of ERα IHC staining with monoclonal antibodies SP1, 6F11, and 1D5. (**A**) SP1 (1:25, Thermo Fisher Scientific, Cat. #: RM-9101-15, Lot:9101513081A), number of cases was 20; (**B**) 6F11 (1:25, Novocastra Laboratories Ltd., Cat. #: NCL-I-ER-6F11, Lot: 6031484), number of cases was 20; and (**C**) 1D5 (1:20, Dako, Cat. #: M7047, lot 1: 00034057, lot 2: 20015818), number of cases was 21.

Significant differences in ERα IHC scores between control tissue and cancer tissue were also observed when using other antibodies against Erα: antibodies SP1 and 6F11 (Figure 2).

### 2.2. Lower mRNA and Protein Levels and Unchanged IHC Scores of ERβ in EC Tissues

**Figure 3:**
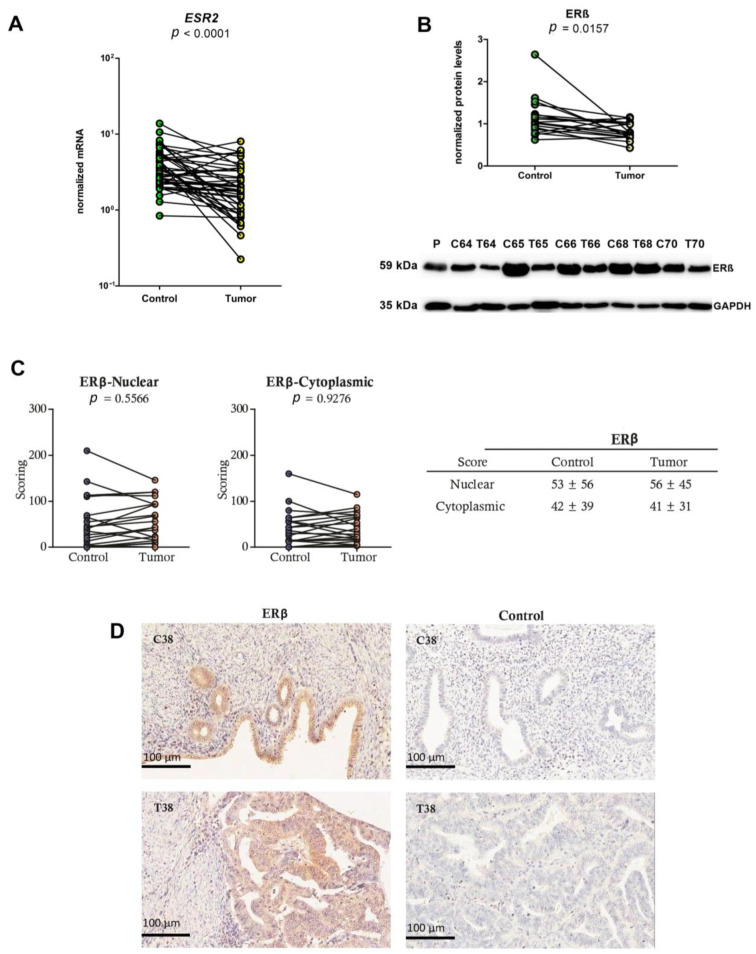
ERβ mRNA and protein levels in endometrial cancer and adjacent control endometrium. (**A**) Before-and-after graph shows the normalized expression levels of the *ESR2* gene in control endometrial tissue (Control) and the corresponding EC tissue (Tumor). The levels of gene expression are on a logarithmic scale. (**B**) ECL detection of ERβ (59 kDa band). 18 paired samples were analyzed using anti-ERβ antibodies (ab3576, Abcam, Cat. #: ab3576, Lot: GR208064-1) and GAPDH was used as a normalization control. Before-and-after plots show quantification of Western blotting data. Below, representative membrane with ERβ and GAPDH staining is shown. EC tissue (T), adjacent control endometrial tissue (**C**), placenta (P) was used as a control tissue. (**C**) IHC scores in 21 samples from adjacent control endometrial tissue (Control) and EC tissue (Tumor). Tables show mean scores ± standard deviations, while before-and-after graphs show nuclear and cytoplasmic ERβ (anti-ERβ antibodies, 14C8, GeneTex, Cat. #: GTX70174, Lot: 20882 (1:100). (**D**) IHC staining in representative paired adjacent control endometrial tissue (C38) and EC tissue (T38) for ERβ. In the negative controls (Control), the primary antibodies were replaced with serum of the same animal species (rabbit, mouse). Anti-ERβ antibodies were validated (Supplementary Figure S6 and Table S5).

Next, we studied ERβ expression at the mRNA and protein levels. The expression of the *ESR2* gene in 44 paired samples of EC tissue and adjacent control endometrial tissue confirmed the previously reported down regulation of *ESR2* in EC tissue [71] (Figure 3A, *p* < 0.0001). 

ERβ protein levels were also investigated using Western blotting (Abcam antibodies, Cambridge, UK, Cat. #: ab3576). A band corresponding to a 59 kDa ERβ protein was detected in all of the samples, and the level of ERβ was significantly lower in the EC compared to adjacent control endometrial tissue (Figure 3B, *p* = 0.0157). Statistical analysis using two-way ANOVA showed that tumor grade does not affect *ESR2* mRNA or protein levels, while menopausal status affects mRNA expression (*p* = 0.037) but not protein levels. Stratification of mRNA data according to menopausal status showed an elevated expression of *ESR2* in control tissue of postmenopausal patients compared to control tissue of premenopausal patients, which significantly accounts for the observed differences in the expression of *ESR2* between control and tumor tissues (Supplementary Figure S5). 

**Figure 4:**
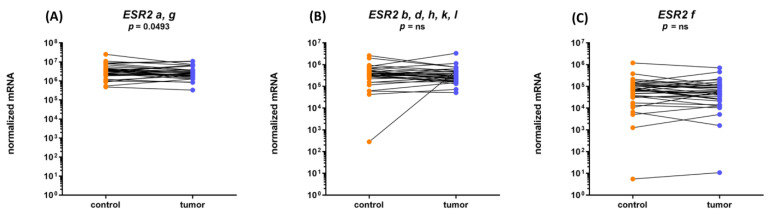
Expression of *ESR2* isoforms in endometrial cancer and adjacent control endometrium. (**A**) *ESR2* isoforms a, g; (**B**) *ESR2* isoforms b, d, h, k, and l; and (**C**) isoform f. Expression in 34 paired EC samples is shown.

Additional analysis of *ESR2* isoforms revealed lower mRNA levels of isoforms a and g in tumor compared to adjacent tissue (*p* = 0.049), while there was no difference in the expression of other *ESR2* isoforms (f, b, d, k, and l) (Figure 4 and Table 1).

In IHC (antibodies 14C8, GeneTex, Irvine, CA, USA), ERβ was detected in the nuclei and cytoplasm of the epithelial cells in the 21 tissue specimens that included both EC tissue and adjacent control endometrial tissue (Figure 3C,D). The nuclear staining of ERβ was noticeably less intense than the staining for ERα (Figure 1C,D and Figure 3C,D), with comparable mean IHC scores for ERβ staining in the nuclei and cytoplasm seen for the EC tissue and the control endometrial tissue (Figure 3C,D). No staining, or very weak staining (IHC score < 50), of the cancerous and the control endometrial glands was observed in 38% and 62% of these samples, respectively. 

### 2.3. Unchanged mRNA Levels and Decreased Protein Levels of GPER in EC Tissue

**Figure 5:**
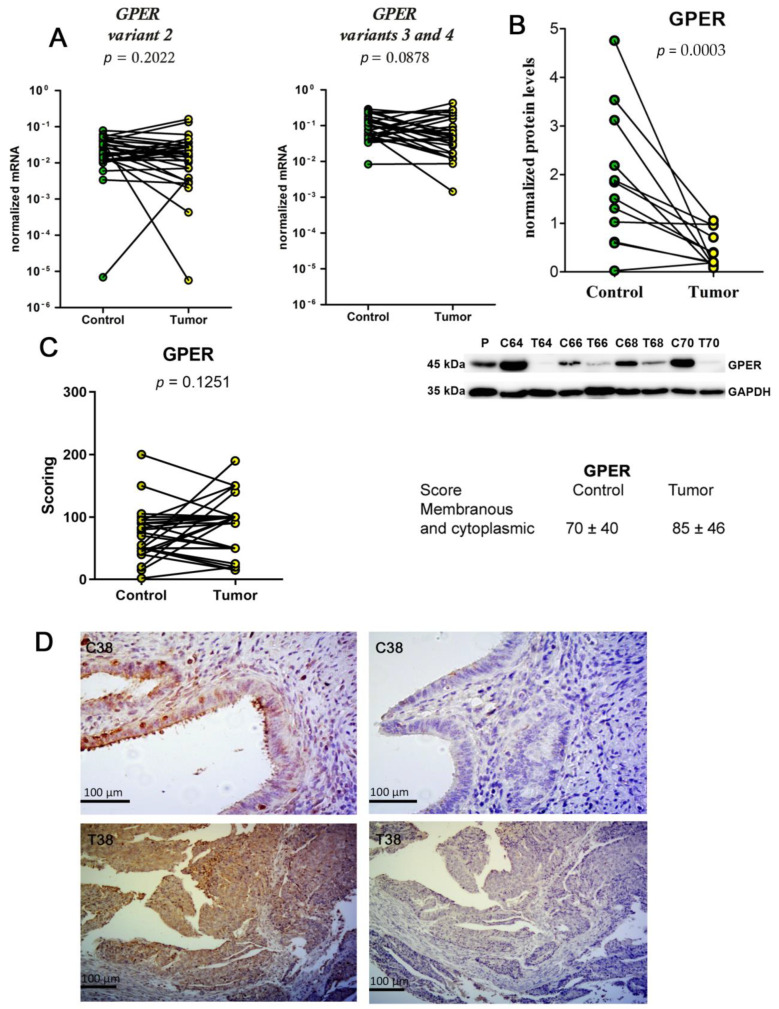
Expression of *GPER* in endometrial cancer tissue and adjacent control endometrium at the mRNA and protein levels. (**A**) Before-and-after graphs show the normalized expression levels of the *GPER* gene variants 2, and variants 3 and 4 (as indicated) in control endometrial tissue (Control) and the corresponding EC tissue (Tumor). The levels of gene expression are on a logarithmic scale. (**B**) ECL detection of GPER. 18 paired samples were analyzed using anti-GPER antibodies (HPA027052, Sigma-Aldrich, Cat. #: HPA027052, Lot: A61748) in the control endometrial tissue (**C**) and EC tissue (T). Placenta (P) was used as the positive control. Below, the detection of GAPDH used for quantification. Before-and-after graph shows the GPER protein levels in the control and EC tissue. (**D**) IHC staining in representative paired adjacent control endometrial tissue (C38) and EC tissue (T38) for GPER. In the negative controls (Control), the primary antibodies were replaced with serum of the same animal species (rabbit). Anti-GPER antibodies were validated by Western blotting analysis (Supplementary Figure S7, Supplementary Table S5).

We examined the *GPER* expression in 31 samples of the EC tissue and the adjacent control endometrial tissue (Figure 5A). We separately amplified *GPER* gene variant 2 (Hs00173506_m1) and variants 3 and 4 (Hs01116133_m1). The expression of *GPER* variant 2 was unchanged in the EC tissue versus the control adjacent endometrial tissue, while the mean expression levels of variants 3 and 4 were decreased 2.4-fold, although this was not statistically significant (Figure 5A). We found a statistically significant correlation between the expression ratios (EC/adjacent control endometrium) of *GPER* variant 2 and variants 3 and 4 (rs = 0.7193; *p* < 0.0001). 

Next, we examined *GPER* expression at the protein level (Sigma-Aldrich, Saint Louis, MO, USA, antibodies, Cat. #: HPA 027052, lot: A61748) with Western blot. A 45 kDa protein was detected in most of the 18 paired tissue samples (Figure 5B). Significantly lower levels of GPER were seen in the EC tissue compared to the adjacent control endometrial tissue (Figure 5C). The two-way ANOVA showed no influence of menopausal status and tumor grade on *GPER* expression at the mRNA or protein levels. 

In IHC (anti-GPER antibodies HPA027052, Cat. #: HPA027052, Lot: A61748 (Sigma-Aldrich, Saint Louis, MO, USA), GPER was detected in the membrane and cytoplasm of the epithelial cells in the 29 tissue specimens that included both EC tissue and adjacent control endometrial tissue (Figure 5D). No staining, or very weak staining (IHC score < 50), of the cancerous and the control adjacent endometrial glands was observed in 24% and 17% of these samples, respectively.

### 2.4. ERα, ERβ, and GPER Are Co-Expressed in EC Tissue and Correlate in Their mRNA and Protein Levels

**Figure 6:**
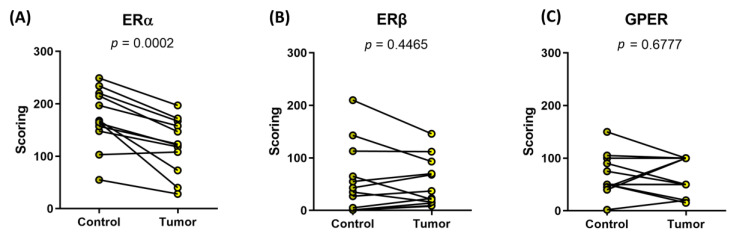
IHC scoring of ERα, ERβ, and GPER staining of the 12 tissue samples from our cohort. Before-and-after graphs show (**A**) ERα (anti-ERα antibodies, 1D5, Dako, Cat. #: M7047, lot 1: 00034057, lot 2: 20015818), (**B**) ERβ (anti-ERβ antibodies, ab3576, Abcam, Cat. #: ab3576, Lot: GR208064-1), and (**C**) GPER (anti-GPER antibodies, HPA027052, Sigma-Aldrich, Cat. #: HPA027052, Lot: A61748) IHC from adjacent control endometrial tissue (Control) and EC tissue (Tumor).

**Figure 7:**
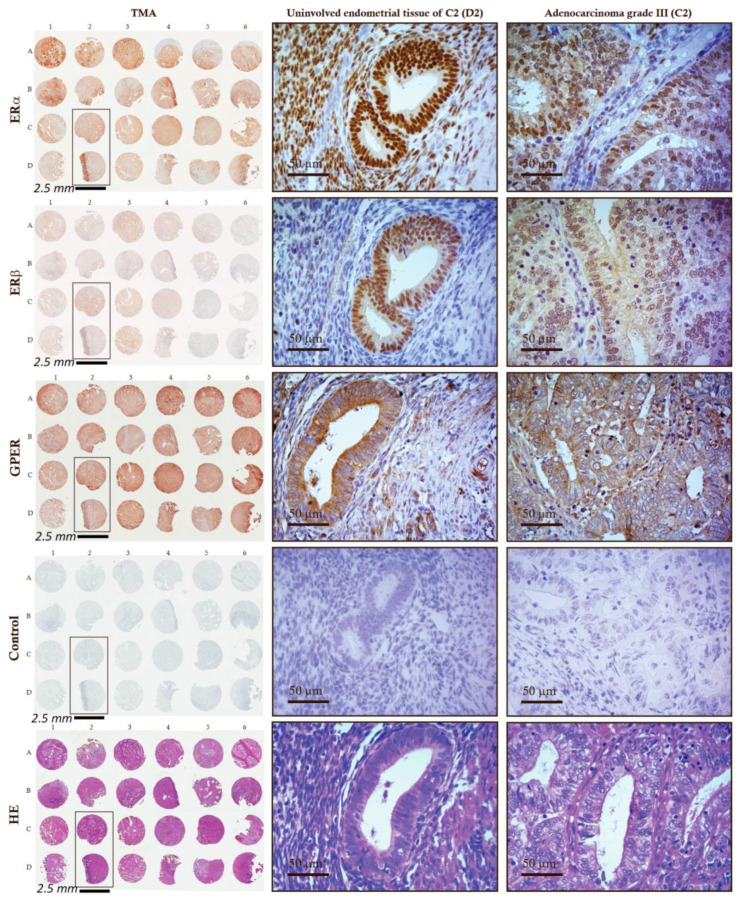
Co-expression of ERα, ERβ, and GPER in EC tissue cores of the tissue microarrays. Positions of samples in tissue microarrays are marked by letters A–D and numbers 1–6. The whole TMAs and control endometrial tissue (D2) and EC tissue (C2) cores are shown for ERα (anti-ERα antibodies, 1D5 Dako, Cat. #: M7047, lot 1: 00034057, lot 2: 20015818), ERβ (anti-ERβ antibodies, 14C8, GeneTex, Cat. #: GTX70174, Lot: 20882), and GPER (anti-GPER antibodies, HPA027052, Sigma-Aldrich, Cat. #:HPA027052, Lot: A61748) staining. The control staining was carried out without the primary antibodies. Sections were also stained with hematoxylin and eosin (HE). Scale bar, 50 µm.

To evaluate co-expression of ERα, ERβ, and GPER in EC tissue, we used samples from our cohort and commercially available TMAs (Figure 6 and Figure 7, Table 2). Our cohort contained 12 paired samples and commercially available TMAs contained 9 paired cores of EC tissue and adjacent control endometrial tissue, respectively. In both cases, the IHC scores for ERα (antibodies 1D5, Dako, Denmark, Cat. #: M7047, lot 1: 00034057, lot 2: 20015818) were significantly lower in the EC compared to the control endometrial tissue (Figure 6: 1A and 2A). ERα was detected in 92% of EC and 100% of control tissue from samples from our cohort and in 67% of EC tissues and 75% of control tissues in the commercial TMAs. In both groups, the IHC reaction was predominantly in nuclei and cytoplasm of the epithelial cells and to a lesser extent in stromal cells of EC tissue. 

In both cohorts, the IHC scores for ERβ (antibodies 14C8, GeneTex, Irvine, CA, USA) were not statistically different in EC compared to adjacent control endometrium (Figure 6: 1B and 2B). In samples from our cohort ERβ was detected in 100% of EC tissue and 75% of control adjacent endometrium and in commercial TMAs it was detected in 67% of EC tissues and 33% of control tissues. In both groups, the IHC reaction was predominantly in nuclei and cytoplasm of the epithelial cells.

The IHC scores for GPER (antibodies HPA027052, Sigma-Aldrich, Saint Louis, MO, USA), were not statistically different in EC compared to adjacent control endometrium in any of the sample groups. In samples from our laboratory, GPER was detected in 100% of EC tissue and 92% of control adjacent endometrium, and in commercial TMAs, it was detected in 100% of EC tissues and 89% of control tissues. In both sample groups, IHC scores for GPER (antibodies HPA027052) were slightly lower in EC, but this was not statistically significant (Figure 6: 1C and 2C). Strong GPER IHC staining was in cytoplasm of epithelial cells and it was also prominent on the luminal side of the cells. In addition to epithelium, the staining was also present in stromal cells, lymphocytes, and smooth muscles (Figure 7).

To examine the co-expression of ERα, ERβ, and GPER in EC and control endometrial tissue, we calculated Spearman’s rank correlation coefficients between expressions of corresponding genes at the mRNA and protein levels and between IHC scores (Table 3). At the mRNA level we found statistically significant correlations between the expression of *ESR1* versus *ESR2*, *ESR1* versus *GPER* (gene variants 3 and 4), and *ESR2* versus *GPER* (variant 3 and 4). The expression of *GPER* (variants 3 and 4) also showed a high correlation with *GPER* (variant 2), but we found no correlation between *GPER* (variant 2) and *ESR1* or *ESR2*. 

At the protein level (Western blotting), we observed statistically significant correlation between ERα and GPER and between ERβ and GPER, whereas there was no correlation between ERα and ERβ. The IHC scores for ERα and ERβ in cytoplasm significantly correlated in our cohort while there was no correlation for nuclear staining, and we did not find correlation between ERα and GPER or ERβ and GPER. In commercial TMAs, we did not find any statistically significant correlations in IHC staining for ERα, ERβ, and GPER, probably due to a very limited number of samples.

### 2.5. IHC Levels of ERα and GPER in Endometrioid EC Are Not Associated with Survival

Patients from our cohort were assigned to the low or high ERα and GPER groups according to the cutoff percentage of IHC-positive tumor cells. We collected survival data and estimated overall survival and disease-free survival. Due to problems with ERβ antibodies, the number of patients with IHC data and survival data was too low for any reliable analyses. The Kaplan–Meier method used in a limited number of patients (35 for ERα and 28 for GPER) did not reveal differences in disease-free survival of patients, but there was a difference in overall survival for patients with ERα above the cutoff value. This was a pilot study, thus, we decided to analyze archival IHC data from patients diagnosed with EC in two consecutive years. In this additional cohort of 139 patients with endometrioid EC, there was no significant difference in overall survival between patients, with a higher percentage of ERα-positive cells versus patients with a lower percentage of ERα-positive cells, but there was a trend of better survival in EC with high ERα levels (above the cutoff value of 80% of positive tumor cells) (Supplementary Figures S8 and S9).

## 3. Discussion

The biological effects of the estrogens are mediated through nuclear ERα and ERβ, and the membrane-bound GPER. Several studies have investigated the roles of these ERs separately, although none have evaluated the expression of all three of these ERs in the same EC tissue samples. 

Our study confirms significantly lower *ESR1* mRNA and protein levels in EC compared to control endometrium by qPCR and Western blotting. The IHC analysis performed in two different sample cohorts (samples from our laboratory and commercial TMA) revealed significantly weaker nuclear staining for ERα in EC tissue compared to adjacent control endometrial tissue. 

When studying ERs, it is important to note that they have several transcripts and splice variants, which are not necessarily detectable with every qPCR assay or antibody (Table 4). Most ERα splice variants [72] are of the exon-skipping variety [13]. According to NCBI [73], the *ESR1* gene has twelve transcript variants encoding five isoforms. According to the manufacturer, our *ESR1* qPCR assay detected six of these transcript variants that encode the isoforms 1, 2, and 4 (Supplementary Table S10). 

For the detection of ERα in Western blotting and IHC analysis, we used three different monoclonal antibodies (Supplementary Table S5), validated by our or other groups (Supplementary Table S5). Two of these (6F11 and SP1) are also routinely used in clinics [74,75]. The anti-ERα antibodies 1D5 used for IHC recognize isoforms 1, 2, 3, and 5 and antibodies SP1 recognize isoforms 1, 2, and 3. Antibodies 6F11 were raised against the whole protein, thus, it is not known which isoforms they recognize. However, published studies [76,77] revealed a high concordance between IHC staining with 6F11 and 1D5 antibodies; our analysis showed decreased ERα levels when using all three antibodies. SP1 anti-ERα antibodies were also used for Western blotting. 

Although our qPCR analysis detected the expression of isoform 1, 2, and 4, while Western blotting and IHC recognized ERα1, ERα2, and ERα3, we saw the same trend of statistically decreased levels of *ESR1* mRNA and ERα protein in cancer tissue compared to adjacent control tissue. This is in line with published reports showing that ERα1 represents the major isoform of ERα [47,71,78].

Lower levels of ERα in EC tissue compared to control endometrial tissue have been reported by others [19,49,50,79,80,81]. The loss of ER suggests deregulation of signaling pathways, whereas mechanisms behind the down regulation of *ESR1* are not unambiguously understood. One explanation, suggested by Sasaki et al. (2003), is that hypermethylation of CpG-enriched regions is an important mechanism of ER loss. They reported a methylated *ESR1*-C promoter in 29 of 32 EC tissue samples, and in none in their normal endometrial tissue [82]. Furthermore, Wang and al. suggested that the loss of transcription factor Forkhead-box A1 (*FOXA1)* is involved in decreased *ESR1* expression during disease progression [51].

ERα levels have been associated with clinicopathological features of EC. Higher ERα levels have been associated with low-grade tumors [19,50,83], while lower levels have been related to high-grade tumors and poor clinical outcome of patients with EC [45,84]. Only few studies reported better disease-free or overall survival in patients with higher ERα IHC levels. This can be explained by use of different cutoff values. Recent studies recommend stratifying EC patients according to the percentage of ERα IHC-positive tumor cells into three groups: high-risk (<10%), intermediate-risk (20–80%), and low-risk (90–100%) groups [53,85]. Our survival analyses, which included a very limited number of patients, reveal differences between patients with high and low IHC staining in overall survival, while additional study using archival clinical data showed only a trend for better survival of endometrioid EC patients with >80% of ERα-positive cells. There was no difference in disease-free survival.

ERβ is believed to oppose the effects of ERα. Our study confirms significantly decreased ERβ mRNA and protein levels in EC tissue compared to control endometrial tissue as determined by qPCR and Western blot analysis. However, IHC analysis revealed no difference in ERβ levels in EC tissue compared to adjacent control endometrial tissue. This discrepancy between the mRNA and protein levels versus IHC levels might be explained by the detection of different splice variants and isoforms by qPCR assays and antibodies used. 

Due to alternative splicing, the *ESR2* gene encodes several transcript variants [86]. The most frequent transcript variants involve changed sequences for exon 8, which results in different C-terminal regions of the translated proteins, whereas other variants are of the exon-skipping variety [13,87]. The *ESR2* gene has seven transcripts that code for five functional proteins (isoforms 1, 2, 3, 5, and 6) and two non-coding transcripts. Our qPCR Taqman assay detected eight mRNA transcripts, encoding isoforms 1, 2, 3, 5, and 6. Primers for individual transcripts have also been designed in our laboratory, and the expression analysis revealed that lower levels of *ESR2* arise from decreased expression of transcripts encoding isoforms 1 and 6. 

The polyclonal anti-ERβ antibodies ab3576 used for Western blotting detect ERβ isoforms 1, 5, and 6. A band of 59 kDa corresponding to the ERβ isoform 1 was detected in all of the tissue samples, with statistically significantly lower levels in the EC tissue than the in control endometrial tissue, which supports our qPCR data. 

The monoclonal 14C8 anti-ERβ antibodies did not work in Western blotting, as previously reported by others [88], and have been used for IHC analysis only. The IHC scores with the 14C8 antibodies did not support the results of Western blotting (ab3576 antibody) and qPCR data, and this can be explained by expression of ERβ in adjacent cells that might have been included in samples analyzed by qPCR and Western blotting.

Additionally, we introduced the well-characterized monoclonal antibodies PPG5/10 that recognize the major ERβ isoform, ERβ1 [89]. Our IHC analysis led to negative staining in control testicular tissue and colon. This highlights the problem with batch-to-batch variations and specificity of antibodies against ERβ, as previously reported [34,35,36,90], leading to irreproducibility of published data. With the IHC analysis recognizing isoforms 1, 2, 3, 5, and 6, we observed no association between the ERβ levels and tumor grade, which is in agreement with Collins et al. (2009), who reported no grade dependence of ERβ1, ERβ2, and ERβ5 expression in EC tissue [50].

To date, the expression profiles and function of the individual ERα and β isoforms are still mostly unclear [91]. Important roles of different ERα and β isoforms have so far been reported in prostate, breast, lung, thyroid, colorectal, ovarian cancer, and metastases [92,93,94,95]. Differences in functional domains of the isoforms affect protein activity [92]. For example, in high-grade ovarian cancer, ERβ1 has an inhibitory role, while both ERβ2 and ERβ5 have been associated with pro-migratory and invasive functions [95]. Individual functional domains of ERs respond to different modulators and degraders, which may have important therapeutic roles in the future [92,93,95]. According to some scientists, understanding the expression levels and functions of individual isoforms represents one of the important challenges in the research of ERs [92].

As ERα and ERβ form functional heterodimers [16], it has been suggested that an imbalance in ERα and ERβ expression might influence endometrial pathogenesis [96]. We detected higher ERα than ERβ mRNA levels in EC compared to control tissue, which is in accordance with published data [50,87,97]. As previously reported [71], we here confirmed no significant changes in the *ESR1*/*ESR2* expression ratio in the EC tissue compared to adjacent control endometrium. The reports on the *ESR2/ESR1* ratio have not always been consistent [98]. Takama et al. [99] reported a significant positive correlation between *ESR2/ESR1* mRNA expression and the depth of myometrial invasion in 36 samples of human EC, but we could not find such a correlation in our samples. Mylonas [46] associated increased ERα/ERβ ratios with ovarian invasion, and Zannoni et al. [45] concluded that the ratios of ERα/ERβ1 and ERα/ERβ2 identify poor clinical outcome for patients with EC, implying prognostic relevance. 

ERα has been associated with better patient prognosis and low-grade EC [46,100,101], whereas the role of ERβ in EC has not been completely elucidated. The loss of ERβ expression is believed to be a common step in estrogen-dependent tumor progression in several cancers, such as breast, ovarian, prostate, and colon cancers [102]. In EC, a decrease in both mRNA and protein ERβ levels (or its isoforms) has also been reported [71,78,80]. Studies also reported that ERβ knockdown could promote cell proliferation by decreasing p21 expression and by increasing Cyclin D1 expression [49]. However, Häring et al. [87] suggest that ERβ has tumor-promoting properties and a potential oncogenic role. Furthermore, steroid hormone receptor expression is a feature of differentiated endometrial cells and lowered receptor levels in EC, including ERβ, could be a sign of diminished cellular differentiation or cellular transformation [27]. Our data here on decreased ERβ protein levels in EC tissue compared to the surrounding control endometrial tissue is in favor of the later hypothesis, while the data on unchanged immunoreactivity do not support these findings.

The rapid and membrane-associated signaling events of E2 can be mediated via the membrane-bound GPER [103]. Our study confirms *GPER* expression in all of the EC tissue samples at the mRNA level and also in most of the samples at the protein levels. We saw no differences in the expression of *GPER* at the mRNA level and protein levels evaluated by IHC, while Western blot analysis showed significantly lower levels in EC. IHC on samples from our cohort and commercial TMA showed predominantly cytoplasmic staining for GPER, with a perinuclear accentuation, which supports its localization in the endoplasmic reticulum and/or cellular membrane, as reported for breast cancer [62]. 

GPER has three different transcript variants, namely, 2, 3, and 4, where all of them encode the same isoform of a protein [104]. In our qPCR approach, we separately amplified transcripts 2, 3, and 4, and for Western blotting and IHC, we used antibodies which recognize products of all three transcripts. Despite this, we observed no difference in mRNA and IHC levels and decreased protein levels by Western blot in EC and adjacent control endometrium tissue. Discrepancies between protein levels evaluated by Western blotting and IHC staining may be explained by GPER expression in cancer cells and also in adjacent stroma in myometrium, as EC samples might have included some stromal and myometrial cells.

To date, only a few studies have been performed evaluating GPER expression in EC. He et al. (2009) [64] reported elevated *GPER* mRNA levels and higher IHC scores in EC. They amplified all three transcripts of *GPER*, although their qPCR analysis included only 10 EC samples, and they compared this *GPER* expression to control endometrium of healthy women [64]. Li et al. demonstrated higher GPER expression in EC tissues than in normal tissue (study included 50 normal endometrium, 52 type I EC, and 47 type II EC) [68]. In contrast, Skrzypczak et al. (2013) [65] reported reduced *GPER* expression in EC tissue compared to premenopausal and postmenopausal endometrial tissue from non-EC patients, but they amplified only transcript variant 4. They found no correlation between *GPER* and *ESR1* expression [65], whereas we observed a statistically significant correlation between *GPER* variants 3 and 4 and *ESR1* and *ESR2* mRNA levels, and also a correlation between GPER, ERα, and ERβ protein levels in our paired samples of EC and adjacent control tissue. Inconsistencies between reported studies and our data probably result from different study designs, samples from separate case and control groups versus paired samples of case group, a relatively low number of samples, and the evaluation of different transcripts.

To date, the role of GPER in EC has not been explained in detail, although the proliferative and invasive effects of GPER have been demonstrated in Ishikawa, RL95-2, HEC-1A, and KLE EC cell lines [64,105,106], which suggests that GPER has an important role in EC pathogenesis. When investigating the potential of GPER as a prognostic and predictive marker in EC, Krakstad et al. (2012) reported that the loss of GPER predicts poor survival and is more common in metastatic lesions compared with primary lesions in ERα-positive EC [66]. Our analysis in a relatively limited number EC patients did not find correlations with the overall or disease-free survival. An important factor when comparing survival analysis are the cutoff values, which can be differently defined; this problem has already been addressed [53,107]. Our study included mainly stage I EC, therefore, we were unable to evaluate correlations between GPER and metastasis, which calls for further studies to be conducted. Recent studies also highlighted the importance of studying additional factors that influence ER signaling, such as other transcription factors, ER binding cofactors [47,108], and chromatin landscape, that are different between, for example, breast and EC cells. This leads to different ER binding profiles and therefore, the expression of different target genes [47,48]. However, those studies are beyond the scope of this article. 

Our study has the following strengths: (1) use of thoroughly validated antibodies; (2) use of several different antibodies against ERα and ERβ; (3) relatively high number of samples analyzed by qPCR and Western blotting; and (4) inclusion of two different populations and sample sources (samples from our cohort and commercial TMA). The weakness of our study is the low number of samples included in the IHC analysis for ERβ and GPER and the low number of high-grade endometrioid tumors. 

## 4. Materials and Methods

### 4.1. Endometrial Tissue

Of the 45 patients who underwent hysterectomies at Department of Gynecology at the University Medical Center Ljubljana, Slovenia and were enrolled in the present study between 2003 and 2010, 14 were premenopausal (mean age, 45.9 ± 7.6 years) and 31 were postmenopausal (mean age, 70.1 ± 8.6 years) (Supplementary Tables S1–S3). The study was approved by the National Medical Ethics Committee of the Republic of Slovenia (0120-701/2017-6). Paired EC tissue and adjacent control endometrial tissue were collected after hysterectomies and immediately placed into RNA Later (Qiagene, Düsseldorf, Germany), an RNA stabilization solution, and kept at −20 °C until RNA extraction. The diagnosis of EC was confirmed histologically by an experienced gynecological pathologist (J.Š. and S.F.G.).

### 4.2. RNA Isolation and qPCR

Total RNA was isolated from the tissue samples using Tri Reagent kits (Sigma-Aldrich, Saint Louis, MO, USA) according to the manufacturer instructions. The quality of the RNA samples was confirmed using an Agilent 2100 Bioanalyzer (Santa Clara, CA, USA), where they showed an average RNA Integrity Number of 7.7. The total RNA was reverse transcribed using SuperScript^®^ VILO^TM^ cDNA synthesis kits (Invitrogen, Carlsbad, CA, USA). One microgram of total RNA was converted into cDNA (20 μL) according to the manufacturer instructions and then stored at −20 °C. *GPER*, *ESR1,* and *ESR2* mRNA expression levels were determined using the exon-spanning hydrolysis probes (FAM dye labeled) that are commercially available as ‘Assay on Demand’ (Applied Biosystems, Foster City, CA, USA). The qPCR analysis for *ESR1* and *ESR2* was performed on 44 paired samples and for *GPER* on 31 paired samples of EC tissue and adjacent control endometrial tissue (Supplementary Table S1). We used a LightCycler 480 Real-Time PCR system (Roche, Basel, Switzerland), with TaqMan Universal PCR Master mix and universal thermocycling parameters recommended by Applied Biosystems (Waltham, MA, USA)*. HPRT1* and *POLR2A* were used as reference genes as described previously [109]. The assays details are shown in Table 5. The gene expression normalization factor for each sample was calculated based on the geometric mean of both of the selected reference genes [110]. The gene expression for each sample was calculated from the crossing point value (Cq) as E^−Cq^, divided by the normalization factor, and multiplied by 10^8^. The Minimum Information for Publication of Quantitative Real-Time PCR Experiments (MIQE) guidelines were considered in the performance and interpretation of the qPCR reactions [111].

The qPCR analysis of *ESR2* isoforms in 34 paired EC samples was performed using SYBR Green I Master (Roche) and primers that were designed in our laboratory (Table 1) as follows: 1st cycle 5 minutes at 95 °C, 45 cycles 10 seconds at 95 °C, 10 seconds at 60 °C, and 21 seconds at 72 °C. The PCR amplification efficiency was determined from the slope of the log-linear portion of the calibration curve for each gene investigated, and this was accounted for in the further calculations. Two reference genes, *POLR2A* in *HPRT1,* were used for normalization. The gene expression for each sample was calculated from the crossing point value (Cp) as E^−Cp^, divided by the normalization factor and multiplied by 10^12^. Results were analyzed with Wilcoxon test and *p* values < 0.05 were considered statistically significant. All data are presented in Supplementary Table S4.

### 4.3. Western Blotting 

Proteins were isolated from 18 paired samples of EC tissue and the adjacent control endometrial tissue (Supplementary Table S2) that had previously been used for the RNA isolation, following the Tri Reagent kit instructions. Protein aliquots of 30 μg were separated by SDS PAGE on 10% Tris-glycine gels. The proteins were transferred from gels to polyvinylidene difluoride membranes (Millipore Corporation, Billerica, MA, USA) and incubated with 5% non-fat milk in Tris Buffered Saline buffer, with 0.1% Tween^®^ 20 (TTBS) for 2 h. 

For the detection of four proteins—ERα, ERβ, GPER, and GAPDH—the membranes were incubated overnight at 4 °C with the primary antibodies. For ERα we used rabbit monoclonal antibodies from Thermo Fisher Scientific, Life Technologies, Waltham, MA, USA, Lot: 9101513081A (1:500, SP1, Cat. #: RM-9101-15, Lot:9101513081A,) in TTBS with 2% non-fat milk powder (Supplementary Figure S1); for ERβ we used the rabbit polyclonal antibodies from Abcam (1:1000, ab3576, Abcam, Cambridge, UK, Cat. #: ab3576, Lot: GR208064-1) in TTBS with 5% non-fat milk powder (Supplementary Figure S2); and for GPER we used anti-GPER rabbit polyclonal antibodies from Sigma-Aldrich (1:500, HPA027052, Sigma-Aldrich, Saint Louis, MO, USA, Cat. #: HPA027052, Lot: A61748) in TTBS with 5% non-fat milk powder (Supplementary Figure S3). The control protein GAPDH was detected with the mouse polyclonal anti-GAPDH antibodies (1:2500, G8795, Sigma-Aldrich, Saint Louis, MO, USA, Cat. #: G8795, Lot: 086K4832) in TTBS with 1% non-fat milk powder. The details of the primary antibodies are provided in the Supplementary Table S5. The polyclonal secondary antibodies were then applied (peroxidase-conjugated goat anti-rabbit IgG + IgM [H + L], 1:4000 Jackson ImmunoResearch Laboratories Inc., West Grove, PA, USA, Cat. #: 111-035-045) for 2 h at 4 °C in TTBS with 1% non-fat milk powder in the case of ERα and ERβ, whereas for GPER, the secondary antibodies were diluted in 3% non-fat milk powder. For GAPDH detection, the membranes were incubated with the secondary antibodies (peroxidase-conjugated IgG + IgM [H + L], 1:5000 Jackson ImmunoResearch Laboratories Inc., West Grove, PA, USA, Cat. #: 111-035-045) for 2 h at 4 °C in TTBS with 1% non-fat milk powder.

Supersignal^TM^ West Pico Chemiluminiscence Substrate (Thermo Fisher Scientific, Life Technologies, Waltham, MA, USA) was used for the detection of the bound antibodies, according to the manufacturer instructions, using a Fujifilm LAS4000 image reader (Fujifilm, Tokyo, Japan). The detection of GAPDH was used as the normalization control. Quantification of the Western blotting was carried out with ImageJ (National Institutes of Health, Bethesda, MD, USA). All data are presented in Supplementary Table S6.

### 4.4. Immunohistochemistry (IHC)

IHC was performed on individual paraffin sections and on tissue microarrays (Supplementary Table S3). Adjacent tissue was available for 29 formalin-fixed, paraffin-embedded endometrial cancer tissue samples. Sections were dewaxed in xylene and rehydrated. Sections were incubated in H_2_O_2_ to block endogenous peroxidase. After antigen retrieval in sodium citrate buffer, the sections were incubated with the monoclonal antibodies, 1D5 anti-ERα (1:20, M7047, Dako, Denmark, Cat. #: M7047, lot 1: 00034057 and lot 2: 20015818) [112] and the 14C8 anti-ERβ antibodies (1:100, GTX70174, GeneTex, Irvine, CA, USA, Cat. #: GTX70174, lot: 20882) [74], and HPA027052 anti-GPER (1:500, Sigma-Aldrich, Saint Louis, MO, USA, Cat. #: HPA027052, Lot: A61748). The peroxidase-antiperoxidase complex with the diaminobenzidine substrate was used to detect the bound antibodies.

IHC staining was performed using commercially available kits and automated staining procedures (BenchMark Ultra, Ventana, Basel, Switzerland) with diaminobenzidine substrate. Two additional anti-ERα antibodies were employed: the monoclonal antibodies 6F11 (1:25, Novocastra Laboratories Ltd., Newcastle upon Tyne, UK, Cat. #: NCL-I-ER-6F11, Lot: 6031484) and SP1 (1:25, Thermo Fisher Scientific, Life Technologies, Waltham, MA, USA, Cat. #: RM-9101-15, Lot: 9101513081A). We also analyzed commercial microarrays including 12 samples of paired EC tissue and uninvolved control endometrial tissue (TMAs; core size, 2.5 mm; EMC241, Pantomics Inc., Fairfield, CA, USA) to detect ERα, ERβ, and GPER with the monoclonal anti-ERα antibodies 1D5 (1:20, M7047, Dako, Denmark, Cat. #: M7047, lot 1: 00034057 and lot 2: 20015818), anti-ERβ monoclonal 14C8 antibodies (1:100, Genetex, Irvine, CA, USA, Cat. #: GTX70174, Lot: 20882), and anti-GPER HPA027052 antibodies (1:500, Sigma-Aldrich, Saint Louis, MO, USA, Cat. #: HPA027052, Lot: A61748). The TMA sections were first evaluated by an experienced gynecological pathologist (S.F.G.) to confirm the diagnoses indicated by the manufacturer. In one tissue pair, both of the sections were diagnosed as tumor sections, while in two other pairs, the normal section was identified as cervical tissue and not control endometrial tissue. These samples were therefore excluded from further evaluation (Supplementary Table S7). The TMA were processed as described above.

Evaluation of the IHC staining levels was performed by (M.H., J.Š., and S.F.G.) based on the percentage of stained cells and the intensity of staining, which were scored as follows: 1, weak; 2, moderate; 3, very strong. The IHC scores were calculated by multiplying the percentages of positive cells (P) by the intensities (I) (Q = P × I; maximum = 300). Both nuclear and cytoplasmic staining were evaluated for ERα: nuclear staining was evaluated for ERβ, while cytoplasmic staining was evaluated for GPER. All data are presented in Supplementary Table S8.

### 4.5. Survival Data 

Survival data were collected for 44 EC patients (Supplementary Table S9). Patients were assigned to the low or high ERα and GPER groups according to the cutoff percentage of IHC-positive tumor cells as estimated by maximally selected rank statistic maxstat R package (0.7–25) R studio version 4.1.3. The Kaplan–Meier method was used to estimate overall survival and disease-free survival. 

Additionally, we collected, from our archives, survival data for all patients treated for endometrioid EC in 2015 and 2016 at Department of Gynecology at University Medical Centre Ljubljana with available IHC ERα data. An overall survival analysis was performed for these 139 patients. 

### 4.6. Statistical Evaluation

The differences in the expression levels of the selected genes were analyzed at the mRNA and protein levels in the EC tissue, as compared to the adjacent control endometrium, using *t*-test or Wilcoxon tests. Two-way ANOVA was performed for the two-factorial comparisons of parameters, depending on the sample (tumor or control) and on the menstrual status (premenopausal or postmenopausal) or on tumor grade. Spearman’s rank correlation coefficients (rs) were used to assess the correlations between the expression ratios of *ESR1*, *ESR2,* and *GPER* at the mRNA and protein levels and between the scores of the immunohistochemistry analysis. Cutoff values for the survival analysis were selected using maximally selected rank statistic maxstat R package (0.7–25) [113]. The Kaplan–Meier analyses were performed to evaluate effects on survival. The statistical calculations and tests were performed using the GraphPad Prism Software for Windows, version 5.00 (San Diego, CA, USA), SPSS software (IBM version 22, Armonk, NY, USA), or R studio version 4.1.3. All of the tests were two-tailed, and differences of *p* < 0.05 are considered as statistically significant.

## 5. Conclusions

To the best of our knowledge, this is the first report of co-expression of ERα, ERβ, and GPER in EC tissue and their correlations at the mRNA and protein level, which suggests that active estrogens formed in EC tissue can have actions through the classical ERs as well as GPER. Correlations in the expression of estrogen receptors suggest that in addition to ERα, ERβ and GPER may also have clinical prognostic value. The co-expression of ERα, ERβ, and GPER, and the precise role of the separate ER isoforms and variants are still not completely elucidated, and thus warrant further studies. 

## Figures and Tables

**Table 1:** Primers for amplification of ESR2 isoforms and reference genes.

Gene	*Forward Primer*	*Reverse Primer*
*HPRT1*	5′ CCTGGCGTCGTGATTAGTG3′	5′TGAGGAATAAACACCCTTTCCA3′
*POLR2A*	5′CAAGTTCAACCAAGCCATTG3′	5′GTGGCAGGTTCTCCAAGG3′
*ESR2 isoforms a, g*	5′GGCATGGAACATCTGCTCAAC3′	5′CACACTGGAGTTCACGCTTC3′
*ESR2 isoform f*	5′TCCTGGTATCCAGTGCATCG3′	5′TTTCATTGCCCACATGCAAGG3′
*ESR2 isoform b, d, h, k, l*	5′GGACTGGGATTGTGTGGTC3′	5′TAGGCATCGGCATTTCCCCT3′

**Table 2:** IHC score and difference between EC and control endometrial tissue.

(**A**) **Our Cohort**	**ERα**	**ERβ**	**GPER**
Control	173 ± 56	58 ± 66	67 ± 39
Tumor	121 ± 52	51 ± 46	63 ± 35
	*p* = 0.0002	*p* = 0.4465	*p* = 0.6777
(**B**) **Commercial tissue microarrays**	**ERα**	**ERβ**	**GPER**
Control	234 ± 96	39 ± 69	151 ± 54
Tumor	73 ± 82	52 ± 70	132 ± 51
	*p* = 0.0078	*p* = 0.8438	*p* = 0.2930

**Table 3:** Correlation between expression of ERα, ERβ, and GPER in EC at the mRNA and protein levels.

	mRNA (qPCR)	Proteins (Western Blotting)	IHC (Our Cohort)	IHC (Commercial TMAs)
**ERα/ERβ**	rs = 0.5124, *p* < 0.0001	rs = 0.2782, *p* = 0.1003	c: rs = 0.4293, *p* = 0.0046	rs = 0.1670, *p* = 0.5079
			n: rs = 0.1059, *p* = 0.5043	
**ERα/GPER**	GPER 2, rs = 0.2374, *p* = 0.0781	rs = 0.6777, *p* < 0.0001	rs = 0.1333, *p* = 0.6860	rs = 0.4563, *p* = 0.0570
	GPER 3,4, rs = 0.4688, *p* = 0.0003			
**ERβ/GPER**	GPER 2, rs = 0.1297, *p* = 0.3406	rs = 0.5598, *p* = 0.0004	rs = 0.1666, *p* = 0.6101	rs = −0.04187, *p* = 0.8690
	GPER 3,4, rs = 0.3375, *p* = 0.0110			
**GPER 2/3,4**	rs = 0.7193, *p* < 0.0001	n/a	n/a	n/a

Spearman’s rank correlation coefficients between expressions of corresponding genes at the mRNA and protein levels and corresponding *p* values.

**Table 4:** Detection of different transcripts and isoforms of ESR1, ESR2, and GPER by qPCR assays and different antibodies.

	Assay	Isoforms		Antibodies	Isoforms
**ERα/*ESR1***	Hs00174860_m1	1, 2 and 4	WB, IHC	SP1	1, 2, 3
			IHC	1D5	1, 2, 3 and 5
			IHC	6F11	n/a
**ERβ/*ESR2***	Hs01100353_m1	1, 2, 3, 5, 6	WB	ab3576	1, 5, 6
			IHC	14C8	1, 2, 3, 5, 6
			IHC (neg.)	PPG5/10	1
**GPER/*GPER***	Hs00173506_m1	1		HPA027052	1
	Hs01116133_m1	1	WB, IHC		

**Table 5:** Assays for the investigated *ESR* and *GPER* genes and reference genes.

Gene	Assay ID	Gene Name
*ESR1*	Hs00174860_m1	Estrogen receptor 1
*ESR2*	Hs01100353_m1	Estrogen receptor 2 (ER beta)
*GPER*	Hs00173506_m1	G-protein–coupled estrogen receptor 1 (GPER) (gene variant 2)
*GPER*	Hs01116133_m1	G-protein–coupled estrogen receptor 1 (GPER) (gene variants 3 and 4)
*HPRT1*	Hs99999909_m1	Hypoxanthine phosphoribosyltransferase 1 (Lesch-Nyhan syndrome)
*POLR2A*	Hs00172187_m1	Polymerase (RNA) II (DNA directed) polypeptide A, 220kDa

## Data Availability

All data are provided in Supplementary Materials.

## Supplementary Materials

The following supporting information can be downloaded at: https://www.mdpi.com/article/10.3390/ijms24033009/s1. References [114,115,116,117,118,119] are cited in the supplementary materials.

## References

[1] Jemal A., Bray F., Center M.M., Ferlay J., Ward E., Forman D. (2011). Global cancer statistics. CA Cancer J. Clin..

[2] Makker V., MacKay H., Ray-Coquard I., Levine D.A., Westin S.N., Aoki D., Oaknin A. (2021). Endometrial cancer. Nat. Rev. Dis. Prim..

[3] Inoue M. (2001). Current molecular aspects of the carcinogenesis of the uterine endometrium. Int. J. Gynecol. Cancer.

[4] Samarnthai N., Hall K., Yeh I.T. (2010). Molecular profiling of endometrial malignancies. Obstet. Gynecol. Int..

[5] Berstein L.M., Tchernobrovkina A.E., Gamajunova V.B., Kovalevskij A.J., Vasilyev D.A., Chepik O.F., Turkevitch E.A., Tsyrlina E.V., Maximov S.J., Ashrafian L.A. (2003). Tumor estrogen content and clinico-morphological and endocrine features of endometrial cancer. J. Cancer Res. Clin. Oncol..

[6] Wan J., Gao Y., Zeng K., Yin Y., Zhao M., Wei J., Chen Q. (2016). The levels of the sex hormones are not different between type 1 and type 2 endometrial cancer. Sci. Rep..

[7] Brinton L.A., Trabert B., Anderson G.L., Falk R.T., Felix A.S., Fuhrman B.J., Gass M.L., Kuller L.H., Pfeiffer R.M., Rohan T.E. (2016). Serum Estrogens and Estrogen Metabolites and Endometrial Cancer Risk among Postmenopausal Women. Cancer Epidemiol. Biomark. Prev..

[8] Sonoda Y., Barakat R.R. (2006). Screening and the prevention of gynecologic cancer: Endometrial cancer. Best Pract. Res. Clin. Obstet. Gynaecol..

[9] Kandoth C., Schultz N., Cherniack A.D., Akbani R., Liu Y., Shen H., Robertson A.G., Pashtan I., Shen R., Benz C.C. (2013). Integrated genomic characterization of endometrial carcinoma. Nature.

[10] Rižner T.L. (2013). Estrogen biosynthesis, phase I and phase II metabolism, and action in endometrial cancer. Mol. Cell. Endocrinol..

[11] Eyster K.M. (2016). The Estrogen Receptors: An Overview from Different Perspectives. Methods Mol. Biol..

[12] Fuentes N., Silveyra P. (2019). Estrogen receptor signaling mechanisms. Adv. Protein Chem. Struct. Biol..

[13] Taylor S.E., Martin-Hirsch P.L., Martin F.L. (2010). Oestrogen receptor splice variants in the pathogenesis of disease. Cancer Lett..

[14] Adlanmerini M., Solinhac R., Abot A., Fabre A., Raymond-Letron I., Guihot A.L., Boudou F., Sautier L., Vessières E., Kim S.H. (2014). Mutation of the palmitoylation site of estrogen receptor α in vivo reveals tissue-specific roles for membrane versus nuclear actions. Proc. Natl. Acad. Sci. USA.

[15] La Rosa P., Pesiri V., Leclercq G., Marino M., Acconcia F. (2012). Palmitoylation regulates 17β-estradiol-induced estrogen receptor-α degradation and transcriptional activity. Mol. Endocrinol..

[16] Matthews J., Gustafsson J.A. (2003). Estrogen signaling: A subtle balance between ER alpha and ER beta. Mol. Interv..

[17] Levin E.R. (2009). Membrane oestrogen receptor alpha signalling to cell functions. J. Physiol..

[18] Luo J., Liu D. (2020). Does GPER Really Function as a G Protein-Coupled Estrogen Receptor. Front. Endocrinol..

[19] Knapp P., Chabowski A., Błachnio-Zabielska A., Walentowicz-Sadłecka M., Grabiec M., Knapp P.A. (2013). Expression of estrogen receptors (α, β), cyclooxygenase-2 and aromatase in normal endometrium and endometrioid cancer of uterus. Adv. Med. Sci..

[20] Prossnitz E.R., Barton M. (2014). Estrogen biology: New insights into GPER function and clinical opportunities. Mol. Cell. Endocrinol..

[21] Wang D., Hu L., Zhang G., Zhang L., Chen C. (2010). G protein-coupled receptor 30 in tumor development. Endocrine.

[22] Soltysik K., Czekaj P. (2013). Membrane estrogen receptors—Is it an alternative way of estrogen action?. J. Physiol. Pharmacol..

[23] Liang J., Shang Y. (2013). Estrogen and cancer. Annu. Rev. Physiol..

[24] Hwang N.M., Stabile L.P. (2021). Estrogen Receptor ß in Cancer: To ß(e) or not to ß(e)?. Endocrinology.

[25] Matthews J., Wihlén B., Tujague M., Wan J., Ström A., Gustafsson J.A. (2006). Estrogen receptor (ER) beta modulates ERalpha-mediated transcriptional activation by altering the recruitment of c-Fos and c-Jun to estrogen-responsive promoters. Mol. Endocrinol..

[26] Lubahn D.B., Moyer J.S., Golding T.S., Couse J.F., Korach K.S., Smithies O. (1993). Alteration of reproductive function but not prenatal sexual development after insertional disruption of the mouse estrogen receptor gene. Proc. Natl. Acad. Sci. USA.

[27] Hapangama D.K., Kamal A.M., Bulmer J.N. (2015). Estrogen receptor β: The guardian of the endometrium. Hum. Reprod. Update.

[28] Ranhotra H.S. (2015). Estrogen-related receptor alpha and cancer: Axis of evil. J. Recept. Signal Transduct. Res..

[29] Jia M., Dahlman-Wright K., Gustafsson J. (2015). Estrogen receptor alpha and beta in health and disease. Best Pract. Res. Clin. Endocrinol. Metab..

[30] Yoriki K., Mori T., Kokabu T., Matsushima H., Umemura S., Tarumi Y., Kitawaki J. (2019). Estrogen-related receptor alpha induces epithelial-mesenchymal transition through cancer-stromal interactions in endometrial cancer. Sci. Rep..

[31] Gustafsson J.A. (2003). What pharmacologists can learn from recent advances in estrogen signalling. Trends Pharmacol. Sci..

[32] Böttner M., Thelen P., Jarry H. (2014). Estrogen receptor beta: Tissue distribution and the still largely enigmatic physiological function. J. Steroid Biochem. Mol. Biol..

[33] Chen G.G., Zeng Q., Tse G.M. (2008). Estrogen and its receptors in cancer. Med. Res. Rev..

[34] Wu X., Subramaniam M., Negron V., Cicek M., Reynolds C., Lingle W.L., Goetz M.P., Ingle J.N., Spelsberg T.C., Hawse J.R. (2012). Development, characterization, and applications of a novel estrogen receptor beta monoclonal antibody. J. Cell. Biochem..

[35] Andersson S., Sundberg M., Pristovsek N., Ibrahim A., Jonsson P., Katona B., Clausson C.M., Zieba A., Ramström M., Söderberg O. (2017). Insufficient antibody validation challenges oestrogen receptor beta research. Nat. Commun..

[36] Hawse J.R., Carter J.M., Aspros K.G.M., Bruinsma E.S., Koepplin J.W., Negron V., Subramaniam M., Ingle J.N., Rech K.L., Goetz M.P. (2020). Optimized immunohistochemical detection of estrogen receptor beta using two validated monoclonal antibodies confirms its expression in normal and malignant breast tissues. Breast Cancer Res. Treat..

[37] Saczko J., Michel O., Chwiłkowska A., Sawicka E., Mączyńska J., Kulbacka J. (2017). Estrogen Receptors in Cell Membranes: Regulation and Signaling. Adv. Anat. Embryol. Cell Biol..

[38] Yang J.Z., O’Flatharta C., Harvey B.J., Thomas W. (2008). Membrane ERalpha-dependent activation of PKCalpha in endometrial cancer cells by estradiol. Steroids.

[39] Tong J.S., Zhang Q.H., Wang Z.B., Li S., Yang C.R., Fu X.Q., Hou Y., Wang Z.Y., Sheng J., Sun Q.Y. (2010). ER-α36, a novel variant of ER-α, mediates estrogen-stimulated proliferation of endometrial carcinoma cells via the PKCδ/ERK pathway. PLoS ONE.

[40] Zhang L., Li X., Zhao L., Zhang G., Wang J., Wei L. (2009). Nongenomic effect of estrogen on the MAPK signaling pathway and calcium influx in endometrial carcinoma cells. J. Cell. Biochem..

[41] Huang T., Zhou J., Wang J. (2022). Calcium and calcium-related proteins in endometrial cancer: Opportunities for pharmacological intervention. Int. J. Biol. Sci..

[42] Saegusa M., Okayasu I. (2000). Changes in expression of estrogen receptors alpha and beta in relation to progesterone receptor and pS2 status in normal and malignant endometrium. Jpn. J. Cancer Res..

[43] Sakaguchi H., Fujimoto J., Aoki I., Toyoki H., Khatun S., Tamaya T. (2002). Expression of oestrogen receptor alpha and beta in uterine endometrial and ovarian cancers. Eur. J. Cancer.

[44] Hu K., Zhong G., He F. (2005). Expression of estrogen receptors ERalpha and ERbeta in endometrial hyperplasia and adenocarcinoma. Int. J. Gynecol. Cancer.

[45] Zannoni G.F., Monterossi G., De Stefano I., Gargini A., Salerno M.G., Farulla I., Travaglia D., Vellone V.G., Scambia G., Gallo D. (2013). The expression ratios of estrogen receptor α (ERα) to estrogen receptor β1 (ERβ1) and ERα to ERβ2 identify poor clinical outcome in endometrioid endometrial cancer. Hum. Pathol..

[46] Mylonas I. (2010). Prognostic significance and clinical importance of estrogen receptor alpha and beta in human endometrioid adenocarcinomas. Oncol. Rep..

[47] Yu K., Huang Z.Y., Xu X.L., Li J., Fu X.W., Deng S.L. (2022). Estrogen Receptor Function: Impact on the Human Endometrium. Front. Endocrinol..

[48] Rodriguez A.C., Blanchard Z., Maurer K.A., Gertz J. (2019). Estrogen Signaling in Endometrial Cancer: A Key Oncogenic Pathway with Several Open Questions. Horm. Cancer.

[49] Hu G., Zhang J., Zhou X., Liu J., Wang Q., Zhang B. (2020). Roles of estrogen receptor α and β in the regulation of proliferation in endometrial carcinoma. Pathol. Res. Pract..

[50] Collins F., MacPherson S., Brown P., Bombail V., Williams A.R., Anderson R.A., Jabbour H.N., Saunders P.T. (2009). Expression of oestrogen receptors, ERalpha, ERbeta, and ERbeta variants, in endometrial cancers and evidence that prostaglandin F may play a role in regulating expression of ERalpha. BMC Cancer.

[51] Wang J., Bao W., Qiu M., Liao Y., Che Q., Yang T., He X., Qiu H., Wan X. (2014). Forkhead-box A1 suppresses the progression of endometrial cancer via crosstalk with estrogen receptor α. Oncol. Rep..

[52] Backes F.J., Walker C.J., Goodfellow P.J., Hade E.M., Agarwal G., Mutch D., Cohn D.E., Suarez A.A. (2016). Estrogen receptor-alpha as a predictive biomarker in endometrioid endometrial cancer. Gynecol. Oncol..

[53] van Weelden W.J., Reijnen C., Küsters-Vandevelde H.V.N., Bulten J., Bult P., Leung S., Visser N.C.M., Santacana M., Bronsert P., Hirschfeld M. (2021). The cutoff for estrogen and progesterone receptor expression in endometrial cancer revisited: A European Network for Individualized Treatment of Endometrial Cancer collaboration study. Hum. Pathol..

[54] Trovik J., Wik E., Werner H.M., Krakstad C., Helland H., Vandenput I., Njolstad T.S., Stefansson I.M., Marcickiewicz J., Tingulstad S. (2013). Hormone receptor loss in endometrial carcinoma curettage predicts lymph node metastasis and poor outcome in prospective multicentre trial. Eur. J. Cancer.

[55] van der Putten L.J.M., Visser N.C.M., van de Vijver K., Santacana M., Bronsert P., Bulten J., Hirschfeld M., Colas E., Gil-Moreno A., Garcia A. (2018). Added Value of Estrogen Receptor, Progesterone Receptor, and L1 Cell Adhesion Molecule Expression to Histology-Based Endometrial Carcinoma Recurrence Prediction Models: An ENITEC Collaboration Study. Int. J. Gynecol. Cancer.

[56] Guan J., Xie L., Luo X., Yang B., Zhang H., Zhu Q., Chen X. (2019). The prognostic significance of estrogen and progesterone receptors in grade I and II endometrioid endometrial adenocarcinoma: Hormone receptors in risk stratification. J. Gynecol. Oncol..

[57] Obata T., Nakamura M., Mizumoto Y., Iizuka T., Ono M., Terakawa J., Daikoku T., Fujiwara H. (2017). Dual expression of immunoreactive estrogen receptor β and p53 is a potential predictor of regional lymph node metastasis and postoperative recurrence in endometrial endometrioid carcinoma. PLoS ONE.

[58] Prossnitz E.R., Oprea T.I., Sklar L.A., Arterburn J.B. (2008). The ins and outs of GPR30: A transmembrane estrogen receptor. J. Steroid Biochem. Mol. Biol..

[59] Bubb M., Beyer A.L., Dasgupta P., Kaemmerer D., Sänger J., Evert K., Wirtz R.M., Schulz S., Lupp A. (2022). Assessment of G Protein-Coupled Oestrogen Receptor Expression in Normal and Neoplastic Human Tissues Using a Novel Rabbit Monoclonal Antibody. Int. J. Mol. Sci..

[60] Vivacqua A., De Marco P., Santolla M.F., Cirillo F., Pellegrino M., Panno M.L., Abonante S., Maggiolini M. (2015). Estrogenic gper signaling regulates mir144 expression in cancer cells and cancer-associated fibroblasts (cafs). Oncotarget.

[61] Cirillo F., Lappano R., Bruno L., Rizzuti B., Grande F., Guzzi R., Briguori S., Miglietta A.M., Nakajima M., Di Martino M.T. (2019). AHR and GPER mediate the stimulatory effects induced by 3-methylcholanthrene in breast cancer cells and cancer-associated fibroblasts (CAFs). J. Exp. Clin. Cancer Res..

[62] Samartzis E.P., Noske A., Meisel A., Varga Z., Fink D., Imesch P. (2014). The G protein-coupled estrogen receptor (GPER) is expressed in two different subcellular localizations reflecting distinct tumor properties in breast cancer. PLoS ONE.

[63] Sjöström M., Hartman L., Grabau D., Fornander T., Malmström P., Nordenskjöld B., Sgroi D.C., Skoog L., Stål O., Leeb-Lundberg L.M. (2014). Lack of G protein-coupled estrogen receptor (GPER) in the plasma membrane is associated with excellent long-term prognosis in breast cancer. Breast Cancer Res. Treat..

[64] He Y.Y., Cai B., Yang Y.X., Liu X.L., Wan X.P. (2009). Estrogenic G protein-coupled receptor 30 signaling is involved in regulation of endometrial carcinoma by promoting proliferation, invasion potential, and interleukin-6 secretion via the MEK/ERK mitogen-activated protein kinase pathway. Cancer Sci..

[65] Skrzypczak M., Schüler S., Lattrich C., Ignatov A., Ortmann O., Treeck O. (2013). G protein-coupled estrogen receptor (GPER) expression in endometrial adenocarcinoma and effect of agonist G-1 on growth of endometrial adenocarcinoma cell lines. Steroids.

[66] Krakstad C., Trovik J., Wik E., Engelsen I.B., Werner H.M., Birkeland E., Raeder M.B., Øyan A.M., Stefansson I.M., Kalland K.H. (2012). Loss of GPER identifies new targets for therapy among a subgroup of ERα-positive endometrial cancer patients with poor outcome. Br. J. Cancer.

[67] Xie B.Y., Lv Q.Y., Ning C.C., Yang B.Y., Shan W.W., Cheng Y.L., Gu C., Luo X.Z., Zhang Z.B., Chen X.J. (2017). TET1-GPER-PI3K/AKT pathway is involved in insulin-driven endometrial cancer cell proliferation. Biochem. Biophys. Res. Commun..

[68] Li Y., Jia Y., Bian Y., Tong H., Qu J., Wang K., Wan X.P. (2019). Autocrine motility factor promotes endometrial cancer progression by targeting GPER-1. Cell Commun. Signal..

[69] Hernández-Silva C.D., Villegas-Pineda J.C., Pereira-Suárez A.L. (2020). Expression and Role of the G Protein-Coupled Estrogen Receptor (GPR30/GPER) in the Development and Immune Response in Female Reproductive Cancers. Front. Endocrinol..

[70] Smuc T., Hevir N., Ribic-Pucelj M., Husen B., Thole H., Rizner T.L. (2009). Disturbed estrogen and progesterone action in ovarian endometriosis. Mol. Cell. Endocrinol..

[71] Smuc T., Rizner T.L. (2009). Aberrant pre-receptor regulation of estrogen and progesterone action in endometrial cancer. Mol. Cell. Endocrinol..

[72] NCBI The National Center for Biotechnology Information. http://www.ncbi.nlm.nih.gov/IEB/Research/Acembly/av.cgi?db=human&term=ESR1.

[73] NCBI The National Center for Biotechnology Information. https://www.ncbi.nlm.nih.gov/gene/2099.

[74] Reisenbichler E.S., Lester S.C., Richardson A.L., Dillon D.A., Ly A., Brock J.E. (2013). Interobserver concordance in implementing the 2010 ASCO/CAP recommendations for reporting ER in breast carcinomas: A demonstration of the difficulties of consistently reporting low levels of ER expression by manual quantification. Am. J. Clin. Pathol..

[75] Cheang M.C., Treaba D.O., Speers C.H., Olivotto I.A., Bajdik C.D., Chia S.K., Goldstein L.C., Gelmon K.A., Huntsman D., Gilks C.B. (2006). Immunohistochemical detection using the new rabbit monoclonal antibody SP1 of estrogen receptor in breast cancer is superior to mouse monoclonal antibody 1D5 in predicting survival. J. Clin. Oncol..

[76] Bevitt D.J., Milton I.D., Piggot N., Henry L., Carter M.J., Toms G.L., Lennard T.W., Westley B., Angus B., Horne C.H. (1997). New monoclonal antibodies to oestrogen and progesterone receptors effective for paraffin section immunohistochemistry. J. Pathol..

[77] Kaplan P.A., Frazier S.R., Loy T.S., Diaz-Arias A.A., Bradley K., Bickel J.T. (2005). 1D5 and 6F11: An immunohistochemical comparison of two monoclonal antibodies for the evaluation of estrogen receptor status in primary breast carcinoma. Am. J. Clin. Pathol..

[78] Paul M., Cholewa K., Mazurek U., Witek A., Wilczok T. (2004). Estrogen receptor beta delta 6 (ER beta delta 6) isoform in human endometrial hyperplasia and adenocarcinoma. Cancer Investig..

[79] Jarzabek K., Koda M., Walentowicz-Sadlecka M., Grabiec M., Laudanski P., Wolczynski S. (2013). Altered expression of ERs, aromatase, and COX2 connected to estrogen action in type 1 endometrial cancer biology. Tumour Biol..

[80] Skrzypczak M., Bieche I., Szymczak S., Tozlu S., Lewandowski S., Girault I., Radwanska K., Szczylik C., Jakowicki J.A., Lidereau R. (2004). Evaluation of mRNA expression of estrogen receptor beta and its isoforms in human normal and neoplastic endometrium. Int. J. Cancer.

[81] Kreizman-Shefer H., Pricop J., Goldman S., Elmalah I., Shalev E. (2014). Distribution of estrogen and progesterone receptors isoforms in endometrial cancer. Diagn. Pathol..

[82] Sasaki M., Kaneuchi M., Fujimoto S., Tanaka Y., Dahiya R. (2003). Hypermethylation can selectively silence multiple promoters of steroid receptors in cancers. Mol. Cell. Endocrinol..

[83] Felix A.S., Stone R.A., Chivukula M., Bowser R., Parwani A.V., Linkov F., Edwards R.P., Weissfeld J.L. (2012). Survival outcomes in endometrial cancer patients are associated with CXCL12 and estrogen receptor expression. Int. J. Cancer.

[84] van Weelden W.J., van der Putten L.J.M., Inda M.A., van Brussel A., Snijders M.P.L.M., Schriever L.M.M., Bulten J., Massuger L.F.A.G., van de Stolpe A., Pijnenborg J.M.A. (2020). Oestrogen receptor pathway activity is associated with outcome in endometrial cancer. Br. J. Cancer.

[85] Weinberger V., Bednarikova M., Hausnerova J., Ovesna P., Vinklerova P., Minar L., Felsinger M., Jandakova E., Cihalova M., Zikan M. (2019). A Novel Approach to Preoperative Risk Stratification in Endometrial Cancer: The Added Value of Immunohistochemical Markers. Front. Oncol..

[86] NCBI The National Center for Biotechnology Information. https://www.ncbi.nlm.nih.gov/gene/2100.

[87] Häring J., Skrzypczak M., Stegerer A., Lattrich C., Weber F., Görse R., Ortmann O., Treeck O. (2012). Estrogen receptor β transcript variants associate with oncogene expression in endometrial cancer. Int. J. Mol. Med..

[88] Shaaban A.M., O’Neill P.A., Foster C.S. (2002). Re: Skliris et al. Evaluation of seven oestrogen receptor beta antibodies for immunohistochemistry, western blotting, and flow cytometry in human breast tissue. J Pathol 2002; 196: 155-162. J. Pathol..

[89] Skliris G.P., Parkes A.T., Limer J.L., Burdall S.E., Carder P.J., Speirs V. (2002). Evaluation of seven oestrogen receptor beta antibodies for immunohistochemistry, western blotting, and flow cytometry in human breast tissue. J. Pathol..

[90] Rizner T.L., Sasano H., Choi M.H., Odermatt A., Adamski J. (2016). Recommendations for description and validation of antibodies for research use. J. Steroid Biochem. Mol. Biol..

[91] Leung Y.K., Mak P., Hassan S., Ho S.M. (2006). Estrogen receptor (ER)-beta isoforms: A key to understanding ER-beta signaling. Proc Natl. Acad. Sci. USA.

[92] Božović A., Mandušić V., Todorović L., Krajnović M. (2021). Estrogen Receptor Beta: The Promising Biomarker and Potential Target in Metastases. Int. J. Mol. Sci..

[93] Gong Z., Yang S., Wei M., Vlantis A.C., Chan J.Y.K., van Hasselt C.A., Li D., Zeng X., Xue L., Tong M.C.F. (2022). The Isoforms of Estrogen Receptor Alpha and Beta in Thyroid Cancer. Front. Oncol..

[94] Pelekanou V., Anastasiou E., Bakogeorgou E., Notas G., Kampa M., Garcia-Milian R., Lavredaki K., Moustou E., Chinari G., Arapantoni P. (2019). Estrogen receptor-alpha isoforms are the main estrogen receptors expressed in non-small cell lung carcinoma. Steroids.

[95] Langdon S.P., Herrington C.S., Hollis R.L., Gourley C. (2020). Estrogen Signaling and Its Potential as a Target for Therapy in Ovarian Cancer. Cancers.

[96] Mylonas I., Makovitzky J., Friese K., Jeschke U. (2009). Immunohistochemical labelling of steroid receptors in normal and malignant human endometrium. Acta Histochem..

[97] Springwald A., Lattrich C., Skrzypczak M., Goerse R., Ortmann O., Treeck O. (2010). Identification of novel transcript variants of estrogen receptor α, β and progesterone receptor gene in human endometrium. Endocrine.

[98] Häring J., Schüler S., Lattrich C., Ortmann O., Treeck O. (2012). Role of estrogen receptor β in gynecological cancer. Gynecol. Oncol..

[99] Takama F., Kanuma T., Wang D., Kagami I., Mizunuma H. (2001). Oestrogen receptor beta expression and depth of myometrial invasion in human endometrial cancer. Br. J. Cancer.

[100] Srijaipracharoen S., Tangjitgamol S., Tanvanich S., Manusirivithaya S., Khunnarong J., Thavaramara T., Leelahakorn S., Pataradool K. (2010). Expression of ER, PR, and Her-2/neu in endometrial cancer: A clinicopathological study. Asian Pac. J. Cancer Prev..

[101] Jongen V., Briët J., de Jong R., ten Hoor K., Boezen M., van der Zee A., Nijman H., Hollema H. (2009). Expression of estrogen receptor-alpha and -beta and progesterone receptor-A and -B in a large cohort of patients with endometrioid endometrial cancer. Gynecol. Oncol..

[102] Bardin A., Boulle N., Lazennec G., Vignon F., Pujol P. (2004). Loss of ERbeta expression as a common step in estrogen-dependent tumor progression. Endocr. Relat. Cancer.

[103] Prossnitz E.R., Barton M. (2011). The G-protein-coupled estrogen receptor GPER in health and disease. Nat. Rev. Endocrinol..

[104] NCBI The National Center for Biotechnology Information. http://www.ncbi.nlm.nih.gov/gene/2852.

[105] Du G.Q., Zhou L., Chen X.Y., Wan X.P., He Y.Y. (2012). The G protein-coupled receptor GPR30 mediates the proliferative and invasive effects induced by hydroxytamoxifen in endometrial cancer cells. Biochem. Biophys. Res. Commun..

[106] Vivacqua A., Bonofiglio D., Recchia A.G., Musti A.M., Picard D., Andò S., Maggiolini M. (2006). The G protein-coupled receptor GPR30 mediates the proliferative effects induced by 17beta-estradiol and hydroxytamoxifen in endometrial cancer cells. Mol. Endocrinol..

[107] van Weelden W.J., Reijnen C., Pijnenborg J.M. (2020). Predictive value of estrogen and progesterone receptors in endometrial hyperplasia and cancer. Acta Obstet. Gynecol. Scand..

[108] Szwarc M.M., Lydon J.P., O’Malley B.W. (2015). Steroid receptor coactivators as therapeutic targets in the female reproductive system. J. Steroid Biochem. Mol. Biol..

[109] Hevir N., Sinkovec J., Rižner T.L. (2011). Disturbed expression of phase I and phase II estrogen-metabolizing enzymes in endometrial cancer: Lower levels of CYP1B1 and increased expression of S-COMT. Mol. Cell. Endocrinol..

[110] Vandesompele J., De Preter K., Pattyn F., Poppe B., Van Roy N., De Paepe A., Speleman F. (2002). Accurate normalization of real-time quantitative RT-PCR data by geometric averaging of multiple internal control genes. Genome Biol..

[111] Bustin S.A., Benes V., Garson J.A., Hellemans J., Huggett J., Kubista M., Mueller R., Nolan T., Pfaffl M.W., Shipley G.L. (2009). The MIQE guidelines: Minimum information for publication of quantitative real-time PCR experiments. Clin. Chem..

[112] Sannino P., Shousha S. (1994). Demonstration of oestrogen receptors in paraffin wax sections of breast carcinoma using the monoclonal antibody 1D5 and microwave oven processing. J. Clin. Pathol..

[113] Hothorn T., Lausen B. (2002). Maxstat: Maximally Selected Rank Statistics.

[114] Welsh A.W., Lannin D.R., Young G.S., Sherman M.E., Figueroa J.D., Henry N.L., Ryden L., Kim C., Love R.R., Schiff R. (2012). Cytoplasmic estrogen receptor in breast cancer. Clin. Cancer Res..

[115] Bogina G., Zamboni G., Sapino A., Bortesi L., Marconi M., Lunardi G., Coati F., Massocco A., Molinaro L., Pegoraro C. (2012). Comparison of anti-estrogen receptor antibodies SP1, 6F11, and 1D5 in breast cancer: Lower 1D5 sensitivity but questionable clinical implications. Am. J. Clin. Pathol..

[116] Hevir N., Trošt N., Debeljak N., Rižner T.L. (2011). Expression of estrogen and progesterone receptors and estrogen metabolizing enzymes in different breast cancer cell lines. Chem. Biol. Interact..

[117] Trošt N., Hevir N., Rižner T.L., Debeljak N. (2013). Correlation between erythropoietin receptor(s) and estrogen and progesterone receptor expression in different breast cancer cell lines. Int. J. Mol. Med..

[118] Flouriot G., Brand H., Denger S., Metivier R., Kos M., Reid G., Sonntag-Buck V., Gannon F. (2000). Identification of a new isoform of the human estrogen receptor-alpha (hER-alpha) that is encoded by distinct transcripts and that is able to repress hER-alpha activation function 1. EMBO J..

[119] Sun J.W., Collins J.M., Ling D., Wang D. (2019). Highly variable expression of ESR1 splice variants in human liver: Implication in the liver gene expression regulation and inter-person variability in drug metabolism and liver related diseases. J. Mol. Genet. Med..

