# Supplementary material for: The Co-Expression of Estrogen Receptors ERα, ERβ, and GPER in Endometrial Cancer
Source: Int J Mol Sci. 2023 Feb 3;24(3):3009. doi: 10.3390/ijms24033009 (PMC9918160; doi:10.3390/ijms24033009)
Supplement: Supplementary file 1 [file ijms-24-03009-s001.zip › ijms-2144782-supplementary.pdf]

Supplement

The Co-Expression of Estrogen Receptors ERα, ERβ, and GPER in Endometrial Cancer

Marko Hojnik [1,2,†], Maša Sinreih [1,†], Maja Anko [1], Neli Hevir-Kene [1], Tamara Knific [1], Boštjan Pirš [1,3], Snježana Frković Grazio [3] and Tea Lanišnik Rižner [1,*]

[1] Institute of Biochemistry and Molecular Genetics, Faculty of Medicine, University of Ljubljana, 1000 Ljubljana, Slovenia
[2] Department of Pathology, University Medical Centre Maribor, 2000 Maribor, Slovenia
[3] Department of Gynecology, University Medical Centre Ljubljana, 1000 Ljubljana, Slovenia
* Correspondence: tea.lanisnik-rizner@mf.uni-lj.si; Tel: +386-1-5437657 Fax: +386-1-5437641
† These authors contributed equally to this work.

Supplementary Table S1. Details of the patients with EC included in qPCR analysis

Sample	Age	Differentiation	Phase	Stage of cancer
Pre-menopausal women				
5[1,2]	39	G2/G3	Secretory	Ib
7[1,2]	50	G1	n/a	n/a
9[1]	41	G1	proliferative	Ia
10[1,2]	53	G1	proliferative	Ia
21[1,2]	53	G2	proliferative	Ia

22[1,2]	36	G1/G2	proliferative	Ia
23[1,2]	45	G1/G2	early secretory	Ia
25[1,2]	54	G3	Proliferative	Ia
30[1,2]	54	G1	early secretory	Ia
32[1,2]	52	G1	Proliferative	n/a
47[1,2]	27	G1/G2	late secretory	Ia
53[1,2]	50	G2/G3	Proliferative	Ia
57[1,2]	43	G1	late secretory	Ia
59[1]	48	G1	Proliferative	n/a
71[1]	48	G1/G2	early secretory	Ia
**Post-menopausal women**				
8[1,2]	83	G2/G3		Ib
11[1,2]	60	G1		Ib
13[1,2]	64	G2		IV
14[1]	73	G2		Ib
16[1]	69	G1/G2		Ia
18[1,2]	79	G1		Ib
19[1,2]	74	G1/G2		Ia
20[1,2]	76	G2/G3		Ia
24[1,2]	69	G2/G3		Ib
26[1,2]	72	G1/G2		Ia
27[1,2]	62	SCE		n/a
33[1]	77	G3		Ib
34[1]	57	G1		Ia
35[2]	61	G2		Ia
38[2]	78	G2/G3		Ia
40[1,2]	71	G1		Ia
42[2]	81	G1		Ia
44[1,2]	73	G1		Ib
46[1]	50	G2/G3		IIIa

49[1]	70	G1		Ia
50[1]	73	G1		Ia
51[1,2]	75	G2/G3		Ia
52[1,2]	75	G2/G3		Ia
54[1,2]	71	G1		Ia
55[1,2]	75	G2		III c
56[1,2]	55	G1/G2		Ia
61[1]	83	G2		Ia
62[1]	59	G1		Ia
63[1]	66	G1/G2		Ia
64[1]	66	G2		Ia
69[1]	80	G1/G2		Ia
70[1]	55	G2		Ib

[1] used in *ESR1* and *ESR2* expression analysis
[2] used in *GPER* expression analysis
G1–G3, grading according to histological differentiation;
SCE, serous adenocarcinoma of endometrium; n/a, data not available.

Supplementary Table S2. Details of the patients with EC included in Western blot analysis

Sample	Age	Differentiation	Phase	Stage of cancer
**Pre-menopausal women**				
7	50	G1	n/a	n/a
21	53	G2	Proliferative	Ia
22	36	G1/G2	Proliferative	Ia
25	54	G3/CC	Proliferative	Ia
53	50	G2/G3	Proliferative	Ia
68	45	G1	late secretory	II
71	48	G1/G2	early secretory	Ia
**Post-menopausal women**				
8	83	G2/G3		Ib

11	60	G1		Ib
49	70	G1		Ia
50	73	G1		Ia
52	75	G2/G3		Ia
54	71	G1		Ia
56	55	G1/G2		Ia
64	66	G2		Ia
65	80	G1/G2		Ib
66	72	G1		Ia
70	55	G2		Ib

G1–G3, grading according to histological differentiation; n/a, data not available.

Supplementary Table S3. Details of the patients with EC included in IHC analysis

**Sample**	**Age**	**Differentiation**	**Phase**	**Stage of cancer**
**Pre-menopausal women**				
5[1,2]	39	G2/G3	Secretory	Ib
9[1,3]	41	G1	Hyperplasia	Ia
10[1,2,3]	53	G1	Proliferative	Ia
21[1,2]	53	G2	Proliferative	Ia
22[1,2,3]	36	G1/G2	Proliferative	Ia
23[1,2,3]	45	G1/G2	early secretory	Ia
30[1,2,3]	54	G1	early secretory	Ia
32[1,2,3]	52	G1	Proliferative	n/a
47[1,3]	27	G3	n/a	Ia
53[1]	50	G2/G3	Proliferative	Ia
57[1,3]	43	G1	late secretory	Ia
59[3]	48	G1	Hyperplasia	n/a
68[1,3]	45	G1	late secretory	II
**Post-menopausal women**				
1[1,3]	n/a	n/a		n/a

2[1,3]	51	G1/G2	Ia
3[1]	65	G1	Ia
4[1]	50	SCE	n/a
6[1]	76	SCE	n/a
8[1,2,3]	83	G2/G3	Ib
11[1,2,3]	60	G1	Ib
13[1,3]	64	G2	IV
19[1,2,3]	74	G1/G2	Ia
20[1,3]	76	G2/G3	Ia
24[1,2,3]	69	G2/G3	Ib
25[1,2]	54	G3	Ia
26[1,2,3]	72	G1/G2	Ia
27[2]	62	SCE	n/a
31[1]	68	G3	Ia
33[1,2]	77	G3	Ib
34[1,2,3]	57	G1	Ia
35[1,2,3]	61	G2	Ia
38[1,2,3]	78	G2/G3	Ia
40[1,2,3]	71	G1	Ia
42[1,2,3]	81	G1	Ia
44[2,3]	73	G1	Ib
46[1,3]	50	G2/G3	IIIa
50[1,3]	73	G1	Ia
52[1,3]	75	G2/G3	Ia
54[1,3]	71	G1	Ia
56[1,3]	55	G1/G2	Ia
58[1,3]	68	G2/G3	Ia
60[1,3]	56	G1	Ia
61[1,3]	83	G2	Ia
62[1,3]	59	G1	Ia
63[1,3]	66	G1/G2	Ia
64[1,3]	66	G2	Ia
65[1,3]	80	G1/G2	Ib
66[1,3]	72	G1	Ia

67[1]	44	G1	Ia
69[1]	72	G1	Ia
70[1]	64	G3	Ib

G1–G3, grading according to histological differentiation.

[1] used in *ERα* expression analysis
[2] used in *ERβ* expression analysis
[3] used in *GPER* expression analysis

Supplementary Table S4. qPCR data

Sample	Tumor *ESR1*	Adjacent control tissue *ESR1*	Tumor; *ESR2*	Adjacent control tissue; *ESR2*	Tumor; *GPER 2*	Adjacent control tissue; *GPER 2*	Tumor; *GPER 3,4*	Adjacent control tissue *GPER 3,4*
5	393.9653	263.2171	7.945704	8.400136	7071.742	6000.935	27586.22	58482.51
7	686.2625	560.5807	37.51868	48.8412	60680.31	78307.39	189173.1	289950.2
8	424.4275	609.0397	9.231734	75.00361	22422.99	10305.79	39745.41	36819.54
9	471.2836	740.1632	24.08998	15.57826	n/a	n/a	n/a	n/a
10	233.1321	1279.272	19.30512	73.77098	3842.602	6.910497	12102.76	53551.62
11	277.0552	481.3439	26.89467	36.77136	22458.66	12123.22	155730.5	40681.38
13	446.3532	1097.303	58.1941	44.0552	27233.47	10140.69	192868.9	53564.65
14	264.1545	168.9907	6.223627	82.53094	n/a	n/a	n/a	n/a
16	194.9453	468.456	11.57568	58.30755	n/a	n/a	n/a	n/a
18	367.394	534.3326	15.99276	23.33428	2941.628	38715.75	11795.85	117689.9
19	765.5381	654.7183	57.93595	106.0864	15328.53	12394.68	93798.68	50857.51
20	572.8148	917.4357	20.56544	23.80673	11388.21	12848.47	75927.85	59746.09
21	405.3151	803.5106	18.13271	20.03128	16034.76	40642.93	46759.18	274061
22	536.4954	1261.504	32.80505	33.17774	35410.76	60708.93	53419.51	94371.73
23	553.0091	330.0548	10.15298	12.84597	37436.32	14276.06	278584.4	71890.2
24	606.1884	965.1884	26.29624	29.70798	10440.41	49925.11	30932.08	159592.9
25	187.8735	375.0709	2.241171	43.5995	429.7693	16720.8	1442.528	59760.43
26	873.0743	829.9862	14.49665	19.2857	28648.71	52072.67	74257.48	177951.7
27	434.0875	549.1291	11.84021	20.59384	46340.96	15066.27	189161.5	76235.09
30	259.3199	486.882	16.91437	33.02182	28363.66	14807.15	40472.73	39532.74

32	1377.245	645.3756	35.80525	26.90524	5.657159	39207.98	232699.4	242192.5
33	447.0218	799.3463	11.56635	53.60176	n/a	n/a	n/a	n/a
34	746.162	1139.078	50.94225	71.55323	n/a	n/a	n/a	n/a
35	n/a	n/a	n/a	n/a	26419.92	14223.93	136190.79	42806.43
38	n/a	n/a	n/a	n/a	39350.01	133817.03	212182.16	271421.72
40	434.3126	863.573	24.78138	21.77211	22040.49	17473.44	56002.33	100452.6
42	n/a	n/a	n/a	n/a	42190.36	27992.67	236489.13	52431.10
44	26.18965	289.8629	8.863353	26.97008	3059.925	22025.94	11768.8	76685.75
46	433.6422	997.6103	15.16306	19.16817	n/a	n/a	n/a	n/a
47	145.142	208.4044	8.456324	36.34069	17736.01	19042.35	16487.82	45523.92
49	512.7081	841.6085	20.81376	25.29042	n/a	n/a	n/a	n/a
50	823.9504	1166.103	41.044	66.56144	n/a	n/a	n/a	n/a
51	580.8589	863.046	56.16735	48.13648	11958.97	11613.01	46837.61	33424.7
52	600.0693	677.6696	8.77013	19.59274	7299.49	27699.49	30071.46	150577.4
53	341.9031	351.3478	17.64279	19.10492	2718.495	3396.233	8704.258	8318.242
54	498.6101	601.2166	6.715055	27.56978	26278.95	29752.99	60674.27	96263.2
55	742.7446	416.4226	19.82302	53.58705	2050.694	14101	17181.86	149845.9
56	380.2175	1803.742	6.086745	43.41941	18312.65	32629.44	43943.09	126662.6
57	1203.531	565.8175	61.36343	22.3036	162230.4	53067.03	432477	121392.7
59	561.3428	493.9125	36.04903	27.8146	7071.742	n/a	n/a	n/a
61	308.9211	1020.794	4.623053	44.49552	60680.31	n/a	n/a	n/a
62	636.9542	1526.204	6.74454	38.82794	22422.99	n/a	n/a	n/a
63	479.8751	1272.401	20.16039	23.20966	n/a	n/a	n/a	n/a
64	1549.355	1515.27	80.30611	50.88978	3842.602	n/a	n/a	n/a
69	416.9694	1695.439	37.31828	138.389	22458.66	n/a	n/a	n/a
70	805.551	2328.816	32.573	48.30988	27233.47	n/a	n/a	n/a
71	344.0704	717.0438	18.31585	26.46525	n/a	n/a	n/a	n/a

Data show normalized mRNA levels for gene expression in endometrial cancer versus adjacent control endometrium. *HPRT1* and *POLR2A* were used as reference genes.

n/a, data not available.

Supplementary Table S5. Data on the antibodies and their validation

**Information on Antibodies**					
**Name of the antibodies**	**Manufacturer, Catalogue #, Batch #,**	**Peptide/ protein target**	**Antigen sequence**	**Species raised, monoclonal, polyclonal, antigen purified**	**Dilution used (mass concentration)**
**SP1**	Thermo Fisher Scientific<br>Cat.: RM-9101-15<br>Lot: 9101513081A	Estrogen receptor α	Synthetic peptide derived from C-terminal of human estrogen receptor. The exact sequence is not known.	Rabbit<br>monoclonal	WB, 1:500<br>IHC, 1:25<br>(home made TMA)
**1D5**	Dako, Denmark<br>Cat.: M7047<br>lot 1: 00034057<br>lot 2: 20015818	Estrogen receptor α	Recombinant human estrogen receptor	Mouse<br>monoclonal	IHC, 1:20 (commercial TMA and tissue samples)
**6F11**	Novocastra reagents<br>Cat.: NCL-I-ER-6F11<br>Lot: 6031484	Estrogen receptor α	Full length recombinant human estrogen receptor molecule	Mouse<br>monoclonal	IHC, 1:25<br>(home made TMA)
**ab3576**	Abcam, Cambridge<br>Cat.: ab3576<br>Lot: GR208064-1	Estrogen receptor β	Synthetic peptide corresponding to rat ERβ aa 467-485, CSSTEDSKNKESSQ NLQSQ	Rabbit<br>polyclonal	WB, 1:1000<br>IHC, 1:50<br>(home made TMA)<br>1 mg/mL
**14C8**	GeneTex<br>Cat.: GTX70174<br>Lot: 20882	Estrogen receptor β	Amino acids 1-153 of human ERβ expressed in E. coli.	Mouse<br>monoclonal	ICH, 1:100, (commercial TMA and tissue samples)<br>1 mg/mL
**PPG5/10**	Serotec<br>Cat.: MCA1974S<br>Lot:290415	Estrogen receptor β	Synthetic peptide corresponding to human ERβ 1 C-terminal region, CEDSKSKEGSQNPQ SQ	Mouse<br>monoclonal	IHC, 1:50<br>(home made TMA)

**PPG5/10**	Bio-Rad<br>Cat.: CA1974ga<br>Lot:180119	Estrogen receptor β	Synthetic peptide sequence CSPAEDSKSKEGSQNPQSQ from the C-terminal region of the human estrogen receptor beta 1 isoform.	Mouse/ IgG2a Monoclonal	IHC: 1:40
**HPA027052**	Sigma Aldrich<br>Cat.: HPA027052<br>Lot: A61748	G-protein coupled estrogen receptor	N-terminal part of GPER, MDVTSQARGVGLEMYPGTAQPAAPNTTSPELNLSHPLLGTALANGTGELSEHQQYVIGLFLS	Rabbit<br>polyclonal	WB, 1:500<br>IHC, 1:500 (commercial TMA, tissue samples, home made TMA)
**GAPDH-71.1**	Sigma Aldrich<br>Cat.: G8795<br>Lot: 086K4832	GAPDH	Rabbit GAPDH	Mouse<br>monoclonal	WB, 1:2500

Validation of antibodies

**Name of the antibodies**	**Western Blotting**		**IHC staining**	**Other methods**<br>**Comparison to mRNA level pattern**
	**positive control**	**negative control**	**as reported previously**	**as reported previously**
**ER*α*, SP1**	endometrium (normal and tumor), cell lines MCF-7, T47D (**Supplementary figure 2)**	Cell lines HEC-1A, MDA-MB-231	[1,2]	Hevir, CBI 2011 [3]
**ER*α*, 1D5**			[1,2]	
**ER*α*, 6F11**			[2]	
**ERβ, Abcam**	Placenta, endometrium	Cell lines MCF10A, HEC-1A		Hevir, CBI 2011[3]

	(normal and tumor) **(Supplementary figure 3)**			
**ERβ, Genetex**			[4]	
**ERβ, Serotec**			[4]	
**GPER, Sigma-Aldrich**	cell lines HEC-1A, Ishikawa, HIEEC, T47-D, MCF-7, MDA-MB-361, placenta, endometrium (normal and tumor) **(Supplementary figure 4)**	human plasma		Trošt, Int J Mol Med 2013 [5]

Supplementary Table S6. Western blot data

Sample	Tumor SP1 ERα	Adjacent control tissue SP1 ERα	Tumor ab3576 ERβ	Adjacent control tissue ab3576 ERβ	Tumor HPA027052 GPER	Adjacent control tissue HPA027052 GPER
7	1.4042998	1.2498437	0.9943709	0.7473072	0.9730671	1.024147
8	1.284748	1.9573406	0.6820519	0.9586733	0.9535498	1.8811284
11	0.1822289	0.8361276	0.7921478	2.6418681	1.0569395	3.5346376
21	0.0228501	1.3053097	0.7781706	1.0314061	0.0838446	2.1876841
22	0.6697362	0.0898636	0.7289429	1.0133621	0.2022414	0.5920459
25	0.806808	2.0728822	0.680776	0.8709613	0.3853598	1.508687
49	0.4158397	1.1240194	0.5794004	0.7767203	0.8170689	1.4861809
50	2.1187236	3.4231334	1.1327562	1.036161	0.6922449	1.6483867
52	0.1569889	0.347271	1.0292599	1.2098785	0.2142434	1.2286067
53	0.0270888	0.0620624	1.0281102	1.1176786	0.5922525	0.9063065
54	0.4602051	1.182892	0.989221	1.1622429	0.4434012	2.1623395
56	0.8471017	1.2784442	0.4306021	0.9153453	0.6531837	1.4623165
64	0.6294953	4.1817924	0.6725186	1.6135238	0.1334212	4.757303

65	0.049444	0.5059976	1.1574506	0.784216	0.1807761	0.6158144
66	1.1362121	1.3826901	1.1417925	1.4582911	0.4013999	1.306845
68	0.7586521	0.3174114	0.7980474	1.2291431	0.7091798	1.8421673
70	0.2749954	2.2285552	0.7225962	1.5288169	0.1273951	3.1215589
71	0.0298683	0.0371818	0.6830975	0.6241894	0.1883572	0.0232325

Data shows normalized protein levels in endometrial cancer versus adjacent control endometrium. GAPDH was used as a loading control.

Supplementary Table S7. Details of commercial tissue array including endometrial cancer tissue and paired adjacent control endometrial tissue and immunohistochemistry scores for ERα, ERβ and GPER

Sample	Age	Pathology	Stage (TNM)	Immunohistochemistry score ERα	ERβ	GPER
A1	44	Adenocarcinoma grade II	T1N0M0	180	190	135
B1	44	Uninvolved endometrial tissue of	-	250	0	135
A2	56	Adenocarcinoma grade II	T1N0M0	200	35	85
B2	56	Uninvolved endometrial tissue of	-	250	120	180
A3	51	Adenocarcinoma grade I-II	T1N0M0	150	147	40
B3	51	Uninvolved endometrial tissue of	-	180	0	25
A4	36	Adenocarcinoma grade II-III	T1N0M0	8	0	120
B4	36	Uninvolved endometrial tissue of	-	300	0	180
A5	40	Adenocarcinoma grade II-III	T2N1M0	40	75	40
B5*	40	Uninvolved endometrial tissue of	-	300	80	200
A6	60	Adenocarcinoma grade II-III	T1N0M0	0	1	135
B6	60	Uninvolved endometrial tissue of	-	0	0	120
C1	49	Adenocarcinoma grade II-III	T1N1M0	1	0	45
D1*	49	Uninvolved endometrial tissue of	-	0	120	213
C2	45	Adenocarcinoma grade III	T1N0M0	55	60	135
D2	45	Uninvolved endometrial tissue of	-	300	190	180
C3**	46	Adenocarcinoma grade III	T3N1M0	90	143	135
D3**	46	Uninvolved endometrial tissue of	-	75	200	135
C4	51	Adenocarcinoma grade III	T3N1M0	0	35	158
D4	51	Uninvolved endometrial tissue of	-	300	40	180
C5	53	Adenocarcinoma grade III	T2N0M0	0	0	158
D5	53	Uninvolved endometrial tissue of	-	285	0	200
C6	56	Adenocarcinoma grade III	T1N0M0	60	0	225
D6	56	Uninvolved endometrial tissue of	-	240	0	160

* These sections were identified as cervix sections, and were excluded from further evaluation.

** Both of these sections were diagnosed as tumor sections, and were excluded from further evaluation.

Supplementary Table S8. Immunohistochemical staining scores* for ERα, ERβ and GPER

Sample	Tumor score; ERα SP1	Tumor score; ERα 6F11	Tumor score; ERα 1D5	Adjacent control tissue score; ERα 1D5	Tumor score; ERβ 14C8	Adjacent control tissue score; ERβ 14C8	Tumor score; GPER HPA027052	Adjacent control tissue score; GPER HPA027052
1	300	300	n/a	n/a	n/a	n/a	100	n/a
2	40	n/a	n/a	n/a	n/a	n/a	40	n/a
3	300	n/a	n/a	n/a	n/a	n/a	n/a	n/a

4	10	0	n/a	n/a	n/a	n/a	n/a	n/a
5	n/a	n/a	218	218	120	113	n/a	n/a
6	2	1	n/a	n/a	n/a	n/a	n/a	n/a
8	160	n/a	160	194	43	13	50	n/a
9	90	n/a	n/a	n/a	n/a	n/a	50	50
10	180	0	167	220	37	27	20	50
11	190	190	121	163	14	1	20	2
13	95	95	n/a	n/a	n/a	n/a	50	n/a
19	n/a	n/a	158	197	23	5	100	45
20	n/a	n/a	n/a	n/a	n/a	n/a	140	20
21	1	0	182	220	120	110	n/a	n/a
22	130	60	197	249	9	1	100	100
23	200	200	172	234	93	143	100	150
24	70	190	118	148	112	113	50	50
25	2	5	110	97	93	40	n/a	n/a
26	40	200	123	157	146	210	50	75
30	60	60	26	60	43	20	100	n/a
31	0	50	n/a	n/a	n/a	n/a	n/a	n/a
32	n/a	n/a	108	103	8	1	15	50
33	n/a	n/a	48	25	56	45	n/a	n/a
34	180	90	16	77	1	1	150	100
35	180	160	28	55	16	35	100	40
38	300	300	73	168	68	43	100	105
40	70	180	40	165	70	55	50	90
42	300	300	147	215	21	65	50	50
44	0	0	60	38	94	69	100	n/a
46	180	180	n/a	n/a	n/a	n/a	50	70
47	190	190	n/a	n/a	n/a	n/a	55	n/a
50	200	300	n/a	n/a	n/a	n/a	90	90
52	300	300	n/a	n/a	n/a	n/a	100	95
53	0	0	n/a	n/a	n/a	n/a	n/a	n/a

54	300	300	n/a	n/a	n/a	n/a	150	95
56	180	300	n/a	n/a	n/a	n/a	25	50
57	180	300	n/a	n/a	n/a	n/a	190	55
58	20	30	n/a	n/a	n/a	n/a	100	45
59	n/a	n/a	n/a	n/a	n/a	n/a	100	100
60	300	300	n/a	n/a	n/a	n/a	100	90
61	50	140	n/a	n/a	n/a	n/a	90	80
62	300	300	n/a	n/a	n/a	n/a	150	200
63	n/a	200	n/a	n/a	n/a	n/a	15	40
64	190	285	n/a	n/a	n/a	n/a	150	50
65	0	0	n/a	n/a	n/a	n/a	50	n/a
66	300	300	n/a	n/a	n/a	n/a	100	83
67	300	300	n/a	n/a	n/a	n/a	n/a	n/a
68	70	70	n/a	n/a	n/a	n/a	135	n/a
69	160	160	n/a	n/a	n/a	n/a	n/a	n/a
70	200	200	n/a	n/a	n/a	n/a	n/a	n/a
71	0	0	n/a	n/a	n/a	n/a	n/a	n/a

*The immunohistochemical scores were calculated by multiplying the percentages of positive cells (P) by the intensities (I) (Q = P × I; maximum = 300).

Supplemtary Table S9. Survival data

Our cohort Sample ID	Percentague of positive tumor cells ERα SP1	Percentague of positive tumor cells GPER HPA027052	Event death	Event disease relapse	Survival (in years since initial diagnosis)	Time to relapse (in years since initial diagnosis)	Type of endometrial cancer
2	40	80	no	no	17.60	16.93	endometrioid
3	100	n/a	no	no	17.30	16.66	endometrioid
7	n/a	n/a	no	no	17.00	16.34	endometrioid
9	45	100	no	no	16.80	16.16	endometrioid
10	90	100	no	no	16.70	16.07	endometrioid
11	90	100	no	no	16.60	15.96	endometrioid
13	95	100	no	no	16.50	15.84	endometrioid

14	n/a	n/a	yes	n/a	14.60	n/a	endometrioid
16	n/a	n/a	yes	n/a	9.80	n/a	endometrioid
18	n/a	n/a	yes	n/a	7.80	n/a	endometrioid
19	10	100	yes	n/a	7.80	n/a	endometrioid
20	100	70	yes	n/a	5.30	n/a	endometrioid
21	1	n/a	yes	n/a	4.00	n/a	endometrioid
22	65	100	no	no	15.70	15.09	endometrioid
23	100	100	no	no	15.50	14.90	endometrioid
24	70	100	yes	yes	2.70	1.32	endometrioid
25	1	n/a	no	no	15.30	14.67	endometrioid
26	40	100	yes	n/a	6.30	n/a	endometrioid
30	30	100	no	no	15.10	14.46	endometrioid
31	0	n/a	yes	n/a	13.40	n/a	endometrioid
33	n/a	n/a	yes	n/a	13.90	n/a	endometrioid
34	90	100	no	no	15.10	14.45	endometrioid
35	90	100	no	no	13.50	12.82	endometrioid
38	100	100	yes	n/a	10.80	n/a	endometrioid
39	n/a	n/a	no	no	13.40	12.79	endometrioid
42	100	100	yes	n/a	12.30	n/a	endometrioid
46	90	100	no	no	12.40	11.78	endometrioid
49	n/a	n/a	no	no	11.80	11.16	endometrioid
50	100	90	no	no	11.80	11.14	endometrioid
51	n/a	n/a	no	no	11.70	11.03	endometrioid
52	100	100	yes	no	11.40	10.99	endometrioid
53	0	n/a	yes	no	11.40	10.96	endometrioid
56	95	50	no	no	11.60	10.95	endometrioid
57	90	95	no	no	11.60	10.91	endometrioid
58	20	100	no	no	11.50	10.86	endometrioid
61	50	90	yes	n/a	9.40	n/a	endometrioid
62	100	100	no	no	10.50	9.83	endometrioid
64	90	100	no	no	10.50	9.81	endometrioid
63	n/a	30	no	no	10.50	9.84	endometrioid
66	100	100	yes	n/a	5.30	n/a	endometrioid
67	100	n/a	no	no	10.30	9.67	endometrioid
68	70	90	no	no	10.20	9.57	endometrioid
69	80	80	no	no	10.20	9.54	endometrioid
70	100	n/a	no	no	10.10	9.50	endometrioid

Additional cohort

**Sample ID**	**Percentague of positive tumor cells ERα SP1**	**Grade**	**Survival (in years since initial diagnosis)**	**Event death**	**Type of endometrial cancer**
a1	100	1	6.92	no	endometrioid
a2	100	1	6.95	no	endometrioid
a3	100	2	6.97	no	endometrioid
a4	100	1	6.99	no	endometrioid
a5	100	2	7.01	no	endometrioid
a6	80	1	7.03	no	endometrioid

a7	95	3	1.55	yes	endometrioid
a8	95	2	5.21	yes	endometrioid
a9	100	1	7.10	no	endometrioid
a10	95	1	7.10	no	endometrioid
a11	100	2	4.24	yes	endometrioid
a12	100	2	7.13	no	endometrioid
a13	100	1	7.14	no	endometrioid
a14	100	1	7.14	no	endometrioid
a15	95	2	7.15	no	endometrioid
a16	20	3	.44	yes	endometrioid
a17	80	1	7.18	no	endometrioid
a18	90	1	7.20	no	endometrioid
a19	100	1	7.24	no	endometrioid
a20	100	1	6.90	yes	endometrioid
a21	100	1	7.30	no	endometrioid
a22	100	2	7.30	no	endometrioid
a23	100	1	7.33	no	endometrioid
a24	100	1	7.33	no	endometrioid
a25	100	1	2.43	yes	endometrioid
a26	95	1	7.37	no	endometrioid
a27	80	1	7.40	no	endometrioid
a28	20	3	2.87	yes	endometrioid
a29	90	3	7.45	no	endometrioid
a30	100	3	7.52	no	endometrioid
a31	100	1	7.56	no	endometrioid
a32	80	2	7.56	no	endometrioid
a33	100	2	7.60	no	endometrioid
a34	90	1	3.17	yes	endometrioid
a35	95	1	7.61	no	endometrioid
a36	90	1	7.62	no	endometrioid
a37	90	1	7.62	no	endometrioid
a38	50	1	7.64	no	endometrioid
a39	100	2	7.64	no	endometrioid
a40	50	2	7.64	no	endometrioid
a41	80	2	3.33	yes	endometrioid
a42	90	1	7.66	no	endometrioid
a43	100	1	7.74	no	endometrioid
a44	95	1	5.00	yes	endometrioid
a45	80	3	7.75	no	endometrioid
a46	100	2	.96	yes	endometrioid
a47	100	1	7.77	no	endometrioid
a48	100	2	7.80	no	endometrioid
a49	100	1	7.80	no	endometrioid
a50	100	2	6.16	yes	endometrioid
a51	95	1	7.80	no	endometrioid
a52	100	1	7.81	no	endometrioid
a53	100	1	7.83	no	endometrioid
a54	80	1	7.89	no	endometrioid
a55	95	3	9.76	no	endometrioid
a56	70	3	9.75	no	endometrioid

a57	100	3	9.55	no	endometrioid
a58	50	3	9.05	no	endometrioid
a59	90	2	8.61	no	endometrioid
a60	100	1	8.36	no	endometrioid
a61	100	1	6.36	yes	endometrioid
a62	95	1	6.81	no	endometrioid
a63	80	2	1.55	yes	endometrioid
a64	80	3	.55	yes	endometrioid
a65	95	2	1.87	yes	endometrioid
a66	95	1	6.74	no	endometrioid
a67	95	1	6.74	no	endometrioid
a68	80	3	.05	yes	endometrioid
a69	100	1	6.68	no	endometrioid
a70	95	2	6.66	no	endometrioid
a71	100	1	3.26	yes	endometrioid
a72	100	1	6.64	no	endometrioid
a73	100	1	6.64	no	endometrioid
a74	100	3	6.61	no	endometrioid
a75	100	1	6.59	no	endometrioid
a76	60	2	2.26	yes	endometrioid
a77	95	2	6.58	no	endometrioid
a78	90	1	1.98	yes	endometrioid
a79	100	1	6.56	no	endometrioid
a80	80	2	6.56	no	endometrioid
a81	40	3	6.56	no	endometrioid
a82	100	1	6.55	no	endometrioid
a83	95	1	1.94	yes	endometrioid
a84	95	1	6.54	no	endometrioid
a85	80	1	6.52	no	endometrioid
a86	95	1	6.52	no	endometrioid
a87	95	1	6.51	no	endometrioid
a88	95	2	6.51	no	endometrioid
a89	100	2	5.37	yes	endometrioid
a90	80	2	6.47	no	endometrioid
a91	100	1	1.59	yes	endometrioid
a92	20	2	6.44	no	endometrioid
a93	90	1	6.44	no	endometrioid
a94	100	1	2.57	yes	endometrioid
a95	95	3	6.43	no	endometrioid
a96	100	2	6.42	no	endometrioid
a97	95	1	3.37	yes	endometrioid
a98	90	1	6.39	no	endometrioid
a99	100	2	6.39	no	endometrioid
a100	90	1	6.38	no	endometrioid
a101	90	1	6.37	no	endometrioid
a102	100	1	6.36	no	endometrioid
a103	100	2	2.53	yes	endometrioid
a104	30	2	6.33	no	endometrioid
a105	100	1	6.30	no	endometrioid
a106	100	1	6.28	no	endometrioid

a107	90	2	6.27	no	endometrioid
a108	100	2	6.27	no	endometrioid
a109	85	1	6.26	no	endometrioid
a110	90	2	6.24	no	endometrioid
a111	100	2	6.24	no	endometrioid
a112	90	1	6.23	no	endometrioid
a113	100	2	6.22	no	endometrioid
a114	95	1	6.21	no	endometrioid
a115	90	2	5.26	yes	endometrioid
a116	80	3	6.16	no	endometrioid
a117	100	1	6.14	no	endometrioid
a118	90	2	6.13	no	endometrioid
a119	95	1	4.21	yes	endometrioid
a120	100	1	6.12	no	endometrioid
a121	95	3	6.10	no	endometrioid
a122	100	2	6.09	no	endometrioid
a123	15	2	6.08	no	endometrioid
a124	90	2	6.08	no	endometrioid
a125	80	3	6.06	no	endometrioid
a126	95	1	6.05	no	endometrioid
a127	80	3	3.61	yes	endometrioid
a128	95	1	5.28	yes	endometrioid
a129	100	2	5.96	no	endometrioid
a130	100	2	5.94	no	endometrioid
a131	90	2	5.94	no	endometrioid
a132	100	2	3.64	yes	endometrioid
a133	100	2	5.92	no	endometrioid
a134	90	1	5.91	no	endometrioid
a135	95	1	5.91	no	endometrioid
a136	95	1	5.89	no	endometrioid
a137	100	1	5.89	no	endometrioid
a138	95	3	5.87	no	endometrioid
a139	80	3	3.31	yes	endometrioid

Supplemtary Table S10. Isoforms of *ESR1* and *ESR2*

ESR1 [6,7]					
mRNA	variant	function	protein	isoform	isoform description
NM_000125.4	variant 1	DNA binding, ligand binding	NP_000116.2	isoform 1	full length ERα
NM_001122740.2	variant 2	DNA binding, ligand binding	NP_001116212.1	isoform 1	full length ERα
NM_001122741.2	variant 3	DNA binding, ligand binding	NP_001116213.1	isoform 1	full length ERα
NM_001122742.2	variant 4	DNA binding, ligand binding	NP_001116214.1	isoform 1	full length ERα

NM_001291230.2	variant 5	DNA binding, ligand binding	NP_001278159.1	isoform 2	full length ERα
NM_001291241.2	variant 6	DNA binding, ligand binding	NP_001278170.1	isoform 3	full length ERα
NM_001328100.2	variant 7	shorter n-term, distinct c-term	NP_001315029.1	isoform 4	truncated ERα without N-terminal AF1 domain that is able to repress ERα activation function ala[6]
NM_001385568.1	variant 8	DNA binding, ligand binding	NP_001372497.1	isoform 1	full length ERα
NM_001385569.1	variant 9	DNA binding, ligand binding	NP_001372498.1	isoform 1	full length ERα
NM_001385570.1	variant 10	DNA binding, ligand binding	NP_001372499.1	isoform 5	distinct C-terminus with preserved DNA binding and ligand binding domains
NM_001385571.1	variant 11	DNA binding, ligand binding	NP_001372500.1	isoform 5	distinct C-terminus with preserved DNA binding and ligand binding domains
NM_001385572.1	variant 12	DNA binding, ligand binding	NP_001372501.1	isoform 5	distinct C-terminus with preserved DNA binding and ligand binding domains
ESR2 [6,7]					

NM_001040275.1	variant B	DNA binding, ligand binding	NP_001035365.1	isoform 2	Shorter and distinct C-terminus
NM_001214902.1	variant d	DNA binding, ligand binding	NP_001201831.1	isoform 3	Shorter and distinct C-terminus
NM_001271876.1	variant f	DNA binding, ligand binding	NP_001258805.1	isoform 5	Shorter and distinct C-terminus
NM_001271877.1	variant g	DNA binding, ligand binding	NP_001258806.1	isoform 6	Lacks an internal segment compared to isoform 1
NM_001291712.2	variant k	DNA binding, ligand binding	NP_001278641.1	isoform 2	Shorter and distinct C-terminus
NM_001291723.1	variant l	DNA binding, ligand binding	NP_001278652.1	isoform 2	Shorter and distinct C-terminus
NM_001437.3	variant a	DNA binding, ligand binding	NP_001428.1	isoform 1	The longest isoform
NR_073496.2	variant h	Non coding			
NR_073497.1	variant i	Non coding			
GPER					
NM_001039966.2	Variant 3	the longest transcript	NP_001035055.1		
NM_001098201.3	Variant 4	differs in the 5' UTR compared to variant 3	NP_001091671.1		
NM_001505.3	Variant 2	differs in the 5' UTR compared to variant 3	NP_001496.1	All variants encode the same protein	

Supplementary Figure S1. Pictures of the whole membranes evaluated by Western blotting analysis with SP1 antibodies against ERα, (ThermoScietific, RM-9101-15).

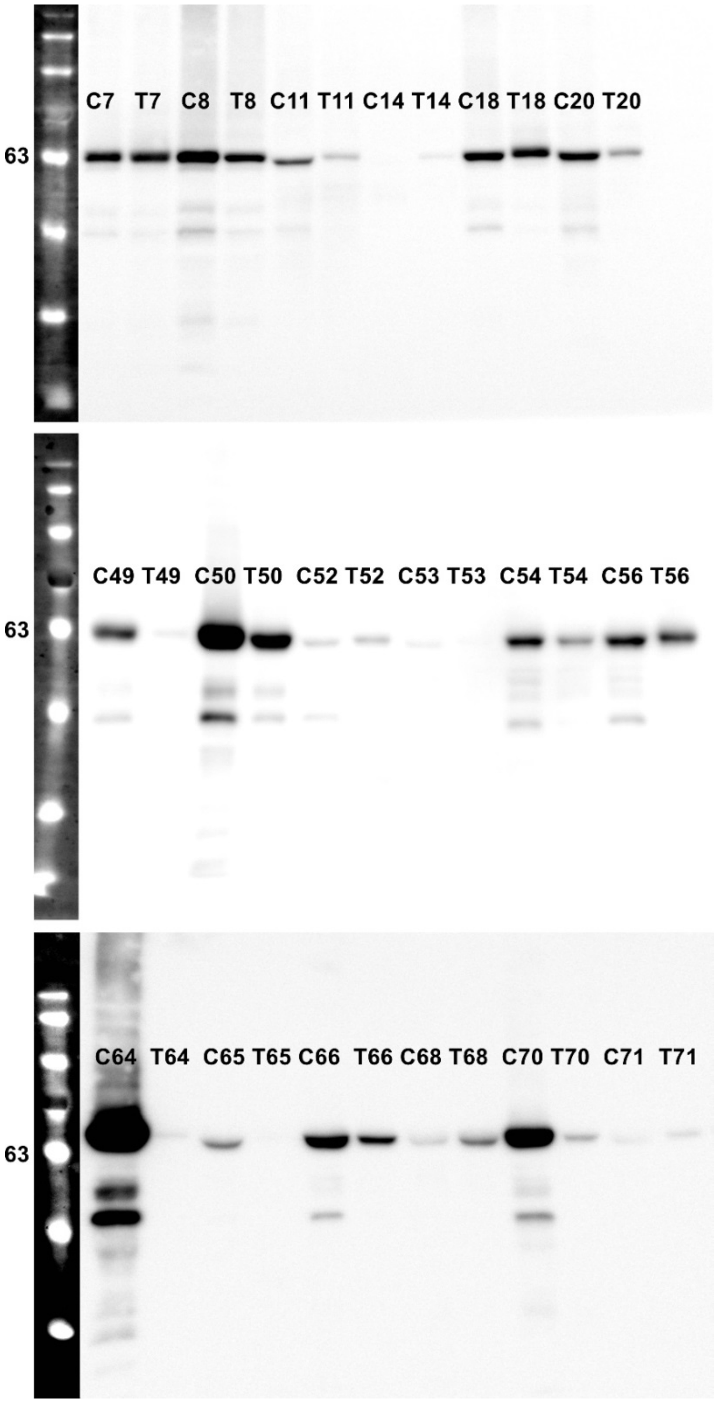

Supplementary Figure S2. Pictures of the whole membranes evaluated by Western blotting analysis using ab3576 (Abcam) antibodies against ERβ.

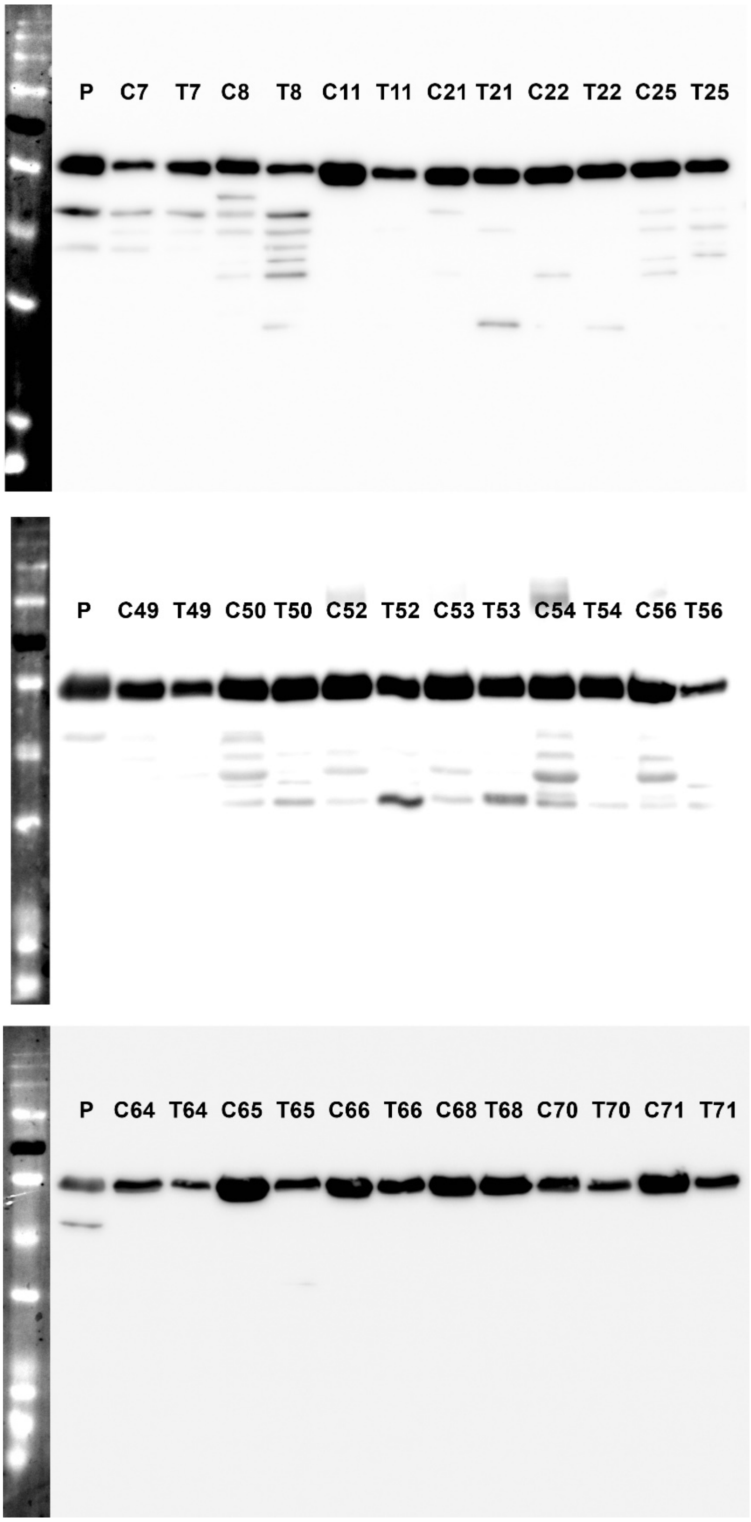

Supplementary Figure S3. Pictures of the whole membranes evaluated by Western blotting analysis using antibodies HPA027052 against GPER (Sigma Aldrich, HPA027052).

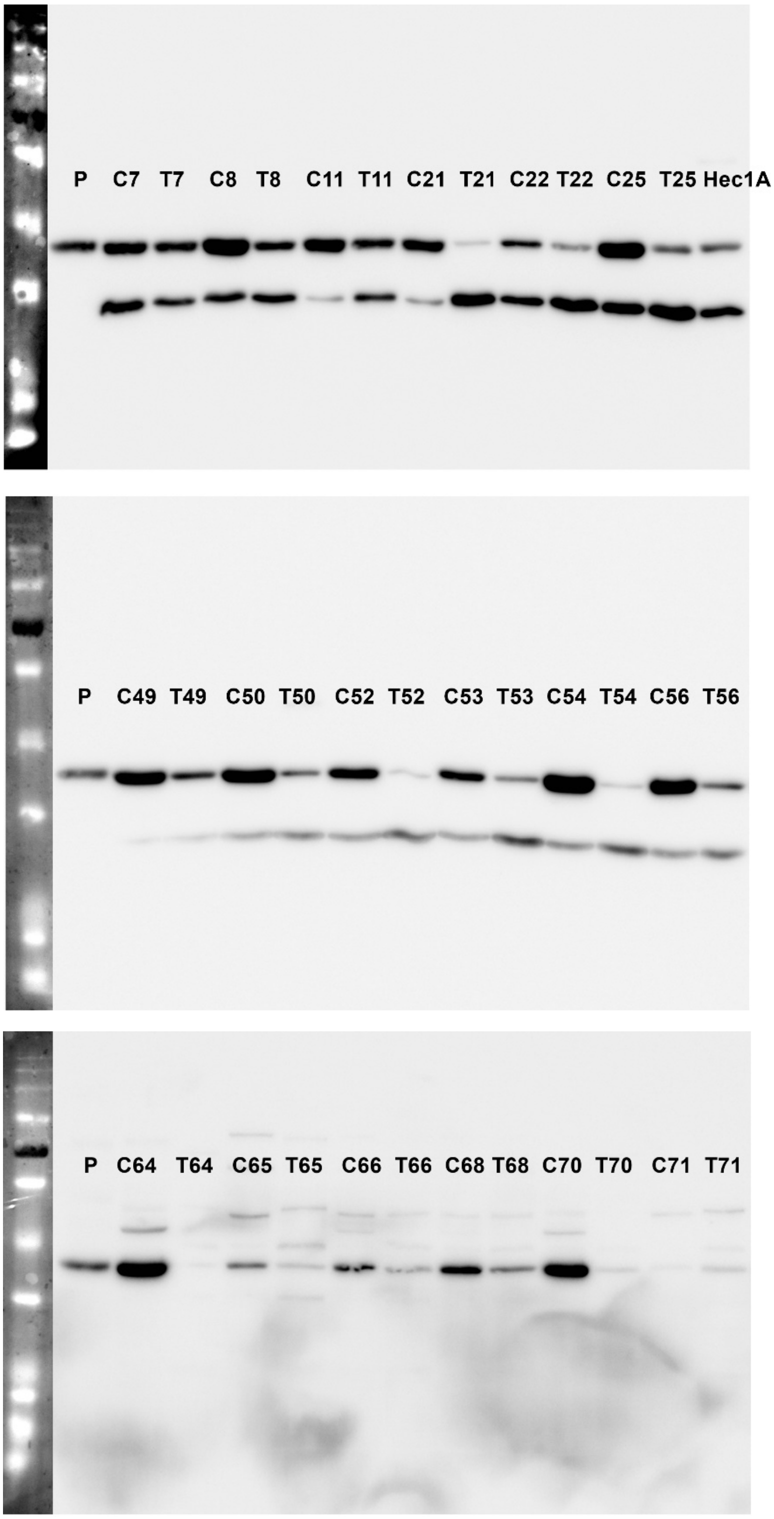

Supplementary Figure S4. Validation for SP-1 antibodies (Thermo Scientific) against ERα.

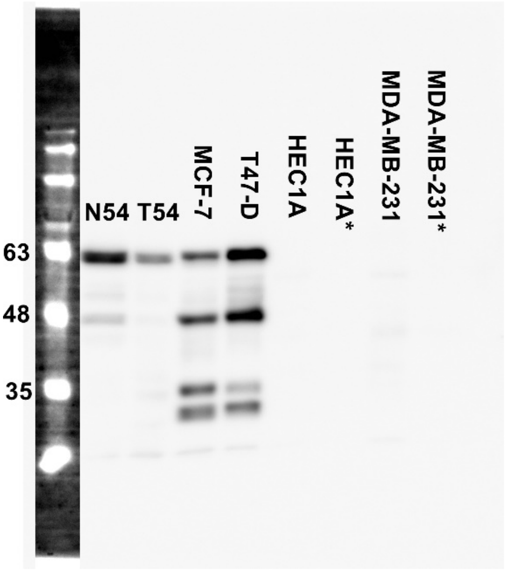

* Protein aliquots of 60 μg; N, tissue of normal endometrium; T, tissue of endometrial cancer

Supplementary Figure S5. Stratification of ESR2 mRNA data according to menopausal status.

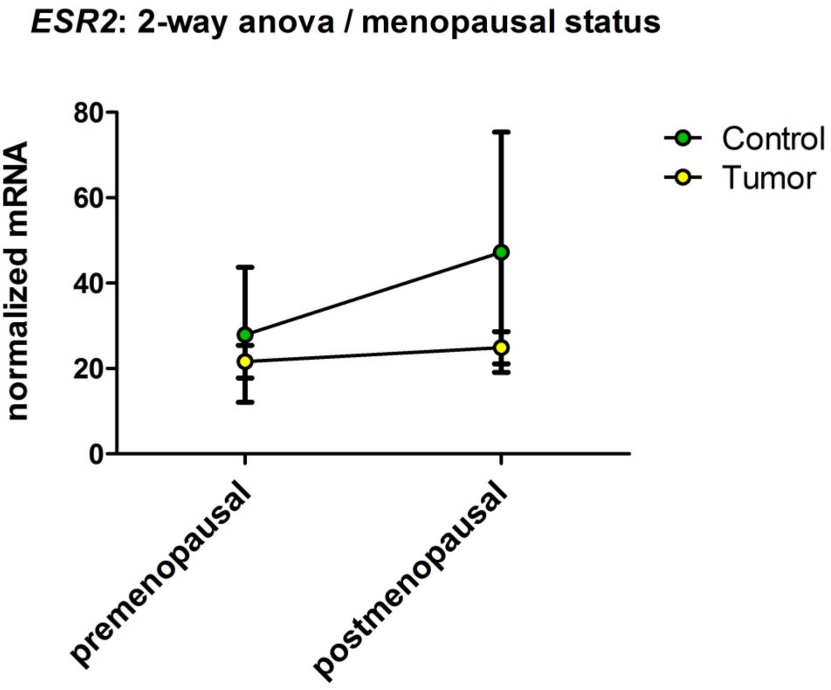

Source of Variation	P value summary	Significant?
Interaction	ns	No
Menopausal status	*	Yes
Disease	**	Yes
Subjects (matching)	*	Yes

Abbreviation: ns, not significant.

*p < .05; **p < .01.

Supplementary Figure S6. Validation for antibodies ab3576 (Abcam) against ERβ**.**

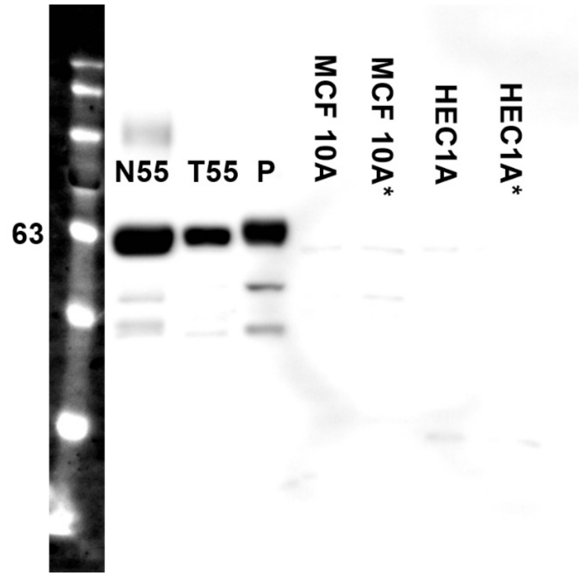

* Protein aliquots of 60 μg; N, tissue of normal endometrium; T, tissue of endometrial cancer; P, placenta tissue

Supplementary Figure S7. Validation for antibodies HPA027052 (Sigma Aldrich) against GPER.

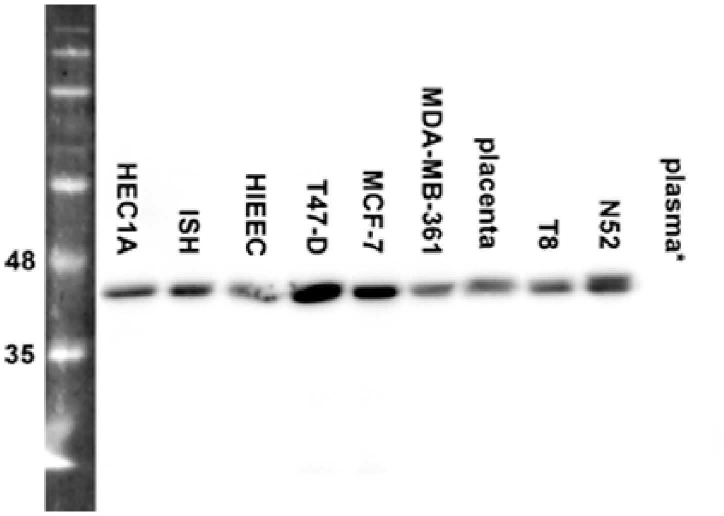

* Protein aliquots of 2 μg; N, tissue of normal endometirum; T, tissue of endometrial cancer

Supplementary Figure S8. Survival curves for patients with endometrioid EC.

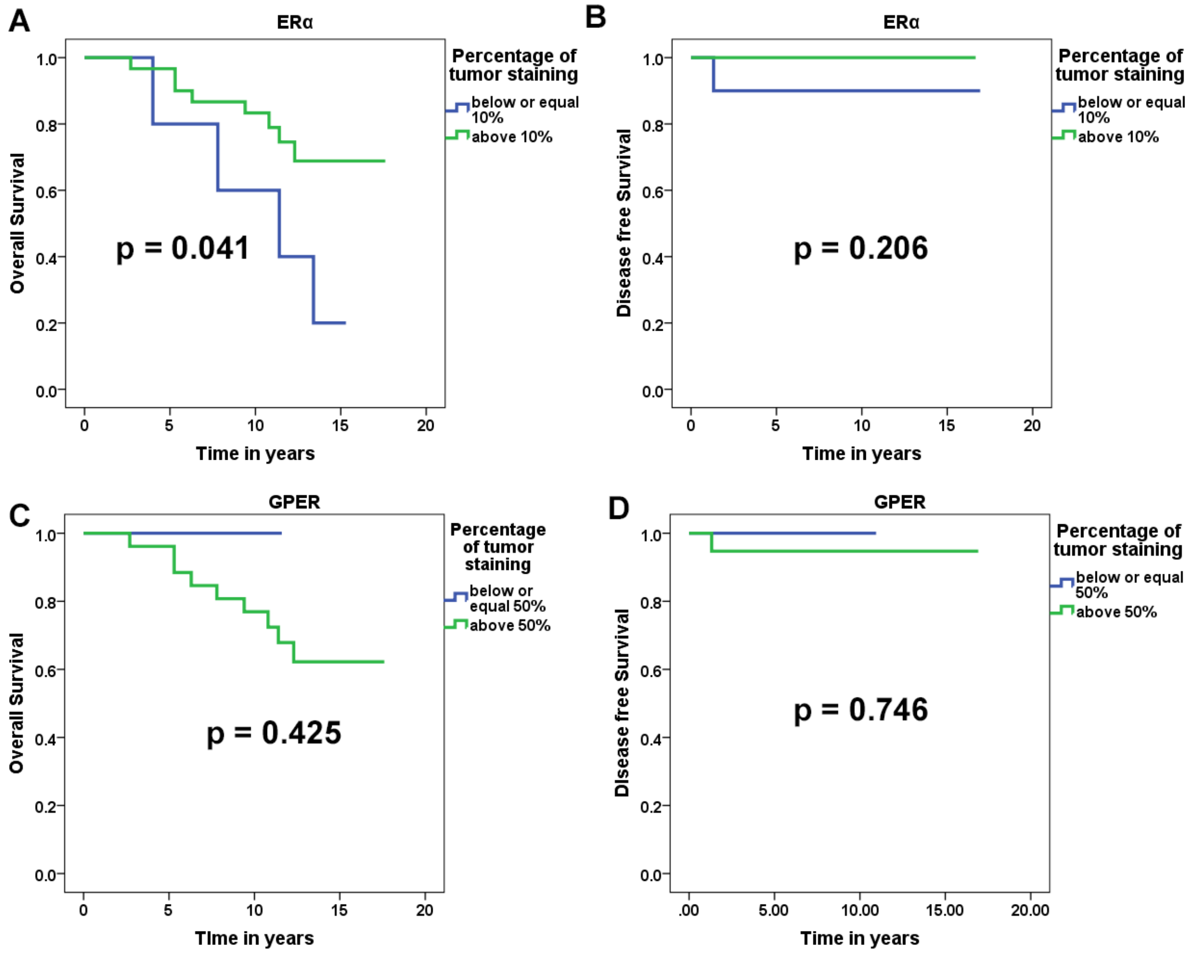

Overall survival and disease-free survival curves for (**A**, **B**) ER*α* and (**C**, **D**) GPER in patients with endometrioid EC. The groups are separated according to their cutoff values for percentage of tumor staining (ER*α* = 10%, GPER = 50%). Number of cases for overall survival for ER*α* was 35 (Grade 1: 22, Grade 2: 9, Grade 3: 4), for GPER was 28 (Grade 1: 20, Grade 2: 8, Grade 3: 0). Number of cases for disease free survival for ER*α* was 26 (Grade 1: 18, Grade 2: 5, Grade 3: 3), for GPER 21 (Grade 1: 16, Grade 2: 5, Grade 3: 0). Time on x-axis represents time elapsed since initial diagnosis.

Supplementary Figure S9. Survival curves for patients with endometrioid EC and comparison with additional cohort

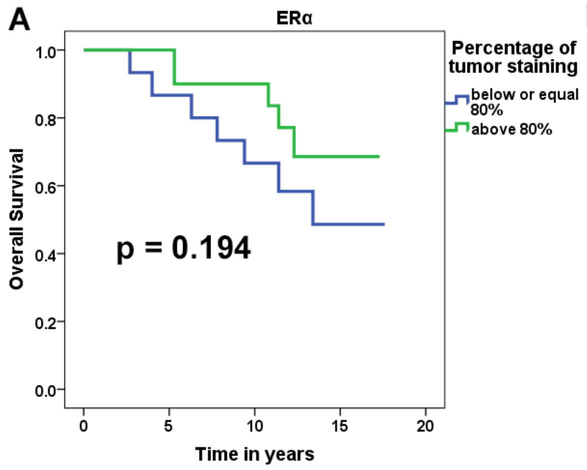

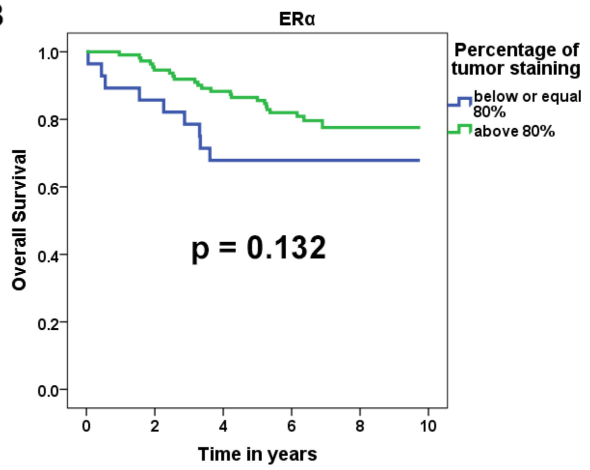

Overall survival curves for ER*α* in (**A**) our cohort of patients with endometrioid EC and in (**B**) additional cohort. The groups are separated according to their cut of values for percentage of positive tumor cells (80%). Number of cases in our cohort was 35 (Grade 1: 22, Grade 2: 9, Grade 3: 4), number of cases in additional cohort was 139 (Grade 1: 73, Grade 2: 45, Grade 3: 21), Time on x-axis represents time elapsed since initial diagnosis.

References:

1. Welsh, A.W.; Lannin, D.R.; Young, G.S.; Sherman, M.E.; Figueroa, J.D.; Henry, N.L.; Ryden, L.; Kim, C.; Love, R.R.; Schiff, R.; et al. Cytoplasmic estrogen receptor in breast cancer. *Clin Cancer Res* **2012**, *18*, 118-126, doi:10.1158/1078-0432.CCR-11-1236.
2. Bogina, G.; Zamboni, G.; Sapino, A.; Bortesi, L.; Marconi, M.; Lunardi, G.; Coati, F.; Massocco, A.; Molinaro, L.; Pegoraro, C.; et al. Comparison of anti-estrogen receptor antibodies SP1, 6F11, and 1D5 in breast cancer: lower 1D5 sensitivity but questionable clinical implications. *Am J Clin Pathol* **2012**, *138*, 697-702, doi:10.1309/AJCPLX0QJROV2IJG.
3. Hevir, N.; Trošt, N.; Debeljak, N.; Rižner, T.L. Expression of estrogen and progesterone receptors and estrogen metabolizing enzymes in different breast cancer cell lines. *Chem Biol Interact* **2011**, *191*, 206-216, doi:10.1016/j.cbi.2010.12.013.
4. Skliris, G.P.; Parkes, A.T.; Limer, J.L.; Burdall, S.E.; Carder, P.J.; Speirs, V. Evaluation of seven oestrogen receptor beta antibodies for immunohistochemistry, western blotting, and flow cytometry in human breast tissue. *J Pathol* **2002**, *197*, 155-162, doi:10.1002/path.1077.
5. Trošt, N.; Hevir, N.; Rižner, T.L.; Debeljak, N. Correlation between erythropoietin receptor(s) and estrogen and progesterone receptor expression in different breast cancer cell lines. *Int J Mol Med* **2013**, *31*, 717-725, doi:10.3892/ijmm.2013.1231.
6. Flouriot, G.; Brand, H.; Denger, S.; Metivier, R.; Kos, M.; Reid, G.; Sonntag-Buck, V.; Gannon, F. Identification of a new isoform of the human estrogen receptor-alpha (hER-alpha) that is encoded by distinct transcripts and that is able to repress hER-alpha activation function 1. *EMBO J* **2000**, *19*, 4688-4700, doi:10.1093/emboj/19.17.4688.
7. Sun, J.W.; Collins, J.M.; Ling, D.; Wang, D. Highly variable expression of ESR1 splice variants in human liver: implication in the liver gene expression regulation and inter-person variability in drug metabolism and liver related diseases.. *J Mol Genet Med* **2019**, *13*.
